# Development of 6-amido-4-aminoisoindolyn-1,3-diones as p70S6K1 inhibitors and potential breast cancer therapeutics

**DOI:** 10.3389/fmolb.2024.1481912

**Published:** 2024-12-19

**Authors:** Adrian Thornton, Rajesh Komati, Hogyoung Kim, Jamiah Myers, Kymmia Petty, Rion Sam, Elijah Johnson-Henderson, Keshunna Reese, Linh Tran, Vaniyambadi Sridhar, Christopher Williams, Jayalakshmi Sridhar

**Affiliations:** ^1^ Department of Chemistry, Xavier University of Louisiana, New Orleans, LA, United States; ^2^ Department of Chemistry, Nicholls State University, Thibodaux, LA, United States; ^3^ Division of Basic Pharmaceutical Sciences, College of Pharmacy, Xavier University of Louisiana, New Orleans, LA, United States; ^4^ Department of Biology, University of New Orleans, New Orleans, LA, United States

**Keywords:** protein kinase, inhibition, potency, breast cancer, docking studies

## Abstract

**Introduction:**

Many breast cancer therapeutics target the PI3K/AKT/mTOR oncogenic pathway. Development of resistance to the therapeutics targeting this pathway is a frequent occurrence. Therapeutics targeting p70S6K1, a downstream member of this pathway, have recently gained importance due to its critical role in all types of breast cancer and its status as a prognostic marker. We have developed a new class of p70S6K1 inhibitors that show growth inhibition of MCF7 breast cancer cells.

**Methods:**

A series of 6-amido-4-aminoisoindolyn-1,3-dione compounds was developed against p70S6K1 using docking, computational modeling tools, and synthesis of the designed compounds. The p70S6K1 inhibition potency of the compounds was investigated in an initial high-throughput screening followed by IC_50_ determination for the most active ones. The best compounds were subjected to proliferation assays on MCF7 breast cancer cells. The targeting of p70S6K1 by the compounds was confirmed by studying the phosphorylation status of downstream protein rpS6.

**Results:**

In this study, we have identified a new class of compounds as p70S6K1 inhibitors that function as growth inhibitors of MCF7 breast cancer cells. The structural features imparting p70S6K1 inhibition potency to the compounds have been mapped. Our studies indicate that substitutions on the phenacetyl group residing in the cleft A of the protein do not contribute to the inhibition potency. Three compounds (**5b, 5d**, and **5f**) have been identified to have sub-micromolar inhibition potency for p70S6K1. These compounds also exhibited growth inhibition of MCF7 cells by 40%–60% in the presence of estradiol.

## Introduction

Downstream signaling molecules of the mTORC1 (mammalian target of rapamycin complex 1) pathway include p70 ribosomal protein S6 kinase 1 (p70S6K1) and eukaryotic translation initiation factor 4E (eIF4E) binding protein 1 (4E-BP1), a regulator of protein synthesis (Lim et al., 2014; Sobral-Leite et al., 2019; Laplante and Sabatini, 2012; Stemke-Hale et al., 2008). They are members of the PI3K/AKT pathway that is frequently activated in several cancers. p70S6K1 plays key roles in migration, invasion, and metastasis, and its overexpression in several types of breast tumors is linked to adverse prognoses for breast cancer (BC) patients (van der Hage et al., 2004; Bahrami et al., 2014). S6K1 belongs to the AGC family of serine/threonine protein kinases. The ribosomal S6 kinase family is comprised of the distinct isoforms: p70S6K1, p85S6K1, p60S6K1, and p31S6K1 (Fenton and Gout, 2011; Magnuson et al., 2012; Tavares et al., 2015; Ben-Hur et al., 2013). These isoforms are encoded through the alternate translational AUG start codons of the *RPS6KB1* gene. p70S6K1 is mostly localized in the cytoplasm, with some studies indicating its accumulation in the nucleus, implying the protein’s transit between the cytoplasm and nucleus (Kim et al., 2009; Coffer and Woodgett, 1994). p70S6K1 is involved in the regulation of several physiological processes, including protein synthesis, differentiation of certain types of cells, ribosomal biogenesis, cell growth, cell survival, cell migration, mRNA processing, translation initiation, elongation, and transcription (Bahrami et al., 2014; Sridhar et al., 2022).

Overexpression of p70S6K1 is present in several types of cancers, including breast, ovarian, prostate, and non-small cell lung cancer (Chen et al., 2017; de Muga et al., 2010; Dobashi et al., 2009; Hussain et al., 2018; Huynh et al., 2017; Ismail, 2012). Expression of p70S6K1 in different types of breast cancers is extensively studied, as the *RPS6KB1* gene is located in the 17q23 chromosomal region that is amplified in 20% of breast cancers (Filonenko et al., 2004; Savinska et al., 2004). p70S6K1 is an established prognostic marker for locoregional recurrence of breast cancer (van der Hage et al., 2004). Overexpression of the receptor tyrosine kinases, such as human epidermal growth factor receptor 2 (HER2), insulin growth factor receptor 1 (IGFR1), and fibroblast growth factor receptor-4, contributes to around 30%–40% of BC, leading to PI3K/AKT pathway and MAPK pathway signaling that converges toward the activation of p70S6K1-assisted translation controlled regulation of cyclin D1 expression (Huynh et al., 2017; Berns et al., 2007; Nahta et al., 2005; Ritter et al., 2007; Scaltriti et al., 2007; Bange et al., 2002). Estrogen receptor (ER) positivity corresponds to 60%–70% of all BC diagnoses, characterized by close interactions between the p70S6K1 and ER signaling. p70S6K1 phosphorylates estrogen receptor alpha (ERα) at residue Ser167 before its translocation to the nucleus for transcriptional activity, leading to enhanced proliferation (Fullwood et al., 2009; Holz, 2012; Maruani et al., 2012; Miller et al., 2011; Yamnik et al., 2009). A positive feedback loop between ERα activation and p70S6K1 levels exists, as estrogen stimulates the expression of p70S6K1. The presence of estrogen is not required for the S6K1 activation of ERα, leading to cell proliferation and tumor transformation. Increased expression of estrogen-related receptor alpha (ERRα), an orphan nuclear factor and a master regulator of cellular energy metabolism, is seen in ERα-negative breast cancer and triple-negative breast cancer (TNBC) (Jarzabek et al., 2009; Stein et al., 2008). Reducing the levels of ERRα in TNBC increased the expression of S6K1 due to increased expression of the *RPS6KB1* gene (Deblois and Giguere, 2013). Amplification of S6K1 (p70S6K1, ribosomal protein S6KB1) and elevated levels of phosphorylated S6K1 are considered biomarkers of aggressive breast cancer (BCa) that are resistant to neoadjuvant chemotherapy (Kim EK. et al., 2013). S6K1 amplification was correlated to innate resistance to CDK4/6 inhibitors in endocrine-resistant BCa, predicting worse survival rates (Mo et al., 2022). S6K1 is identified as a critical target for radio-sensitization in breast cancer patients, as elevated p-S6K1 levels were characterized as a marker for resistance to radiation therapy (Choi et al., 2020). S6K1 inhibition is a viable target for therapeutic interventions, with great potential for resistant/metastatic/refractory cancers to re-sensitize cancer cells to current therapeutics, and a combination therapy would lead to better outcomes for BCa.

**FIGURE 1:**
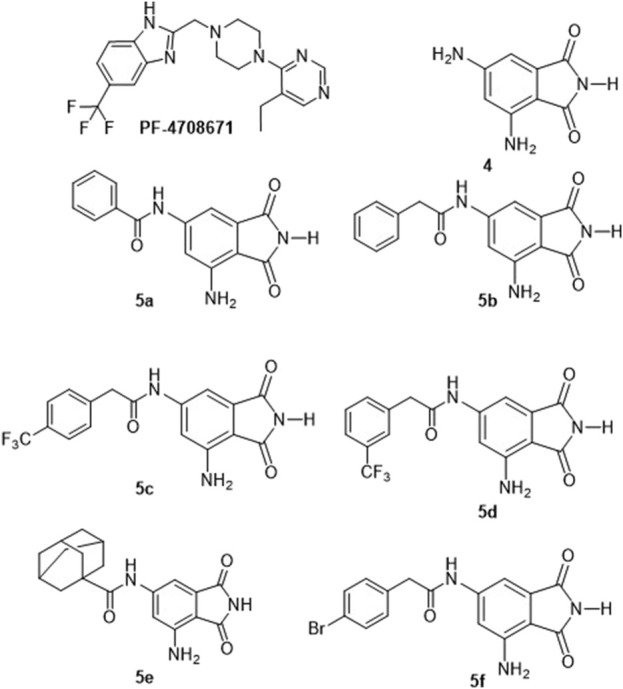
Structures of the identified p70S6K1 inhibitors. PF-4708671 is a known selective inhibitor of p70S6K1. Compounds **4**, **5a–5f** were synthesized.

The prominence of p70S6K1 activity in breast cancer advancement has led to increased interest in the development of inhibitors for this kinase. Derivatives of several classes of compounds, including indazoles, imidazoles, benzimidazoles, ureas, thiones, phenylpyrazoles, pyrolopyrimidines, and organometallics, have shown inhibition activity against p70S6K1. Although many of them exhibited low micromolar inhibition potency, a select few of them had nanomolar potency, such as PF-4708671, FS-115, and FL772, with IC_50_ values of 0.160 μM, 0.035 μM, and 0.0073 μM, respectively (Pearce et al., 2010; Qin et al., 2015; Segatto et al., 2016). PF-4708671 is an S6K1-specific inhibitor used as the standard investigative agent in studies exploring the role of S6K1 in cellular mechanisms (Choi et al., 2013; Hong et al., 2013; Segatto et al., 2013). PF-4708671 was well-tolerated and showed great promise in preclinical studies for breast cancer but was a poor blood–brain barrier (BBB) penetrant (Pearce et al., 2010; Segatto et al., 2016). Another selective S6K1 inhibitor, LY-2584702, the tosylate form of LYS6K2, was not well-tolerated in Phase I trials when given with erlotinib and everolimus (Li et al., 2010; Motzer et al., 2015; Tolcher et al., 2014). Rosmarinic acid methyl ester, a natural compound and potent but non-selective inhibitor of S6K1 when used for the treatment of cervical cancer cells, showed an increase in autophagy and apoptotic cell death (Nam et al., 2019). Our laboratory has identified a new class of compounds, derivatives of 6-amido-4-aminoisoindolyne-1,3-dione (Figure 1), that can provide a class of inhibitors with differing pharmacokinetic and pharmacodynamics properties than the reported S6K1 inhibitors. Our new series of compounds exhibit inhibition of p70S6K1 with nanomolar IC_50_ values in a comparable range (0.4–0.75 μM) to that of PF-4708671 (0.17 μM). The use of computational molecular modeling tools assisted in the design of derivatives with improved potency. p70S6K1 has the classical conserved structural core of protein kinases with two domains: the N-terminal domain that has a 5-stranded β–sheet and the C-terminal domain that is mostly α–helices with a β–sheet that plays a critical role in the kinase activity. The ATP-binding cavity, located along the hinge region connecting the two domains, is made up of the key hydrogen bonding residues Glu173 and Leu175 between strands β1 and β2, with hydrophobic residues lining the top (Leu97 and Ala121) and bottom (Met225), and with other hydrophobic residues Leu172 and Val105 flanking the inner and outer edges close to the hinge region. Outreach to extended regions of the ATP-binding pocket includes the ATP-phosphate binding site, which is occupied by Lys241, the cleft A (activation loop cleft) that is bordered by the β1-P-loop-β2 moiety, and the residues of the activation loop (A-loop). The X-ray crystal structure of PF-4708671 (3WE4.pdb) was used for the design of new derivatives using the lead structure of compound **4**. The design and development of this class of inhibitors is described here.

## Materials and methods

Technical-grade solvents for extraction and chromatography (hexane, cyclohexane, dichloromethane, and ethyl acetate) were used without purification. All reagents were purchased from standard suppliers (Sigma-Aldrich, Alfa Aesar, and Fisher Scientific). Starting materials, if commercial, were purchased and used as such, provided that adequate checks (NMR) had confirmed the claimed purity. The reactions were monitored by thin-layer chromatography (TLC). Aluminum-backed 60 F254 silica plates were used for thin-layer chromatography. Flash chromatography was performed using the Teledyne Isco CombiFlash automated column machine. NMR data were collected on an Agilent 400 MHz instrument. The chemical shifts were reported in parts per million (ppm) relative to the deuterated solvent DMSO-d6; that is, δ ^1^H, 2.49 ppm; ^13^C, 39.7 ppm. High-resolution mass spectral (HRMS) analyses were performed on a Thermo LTQ-Orbitrap LC/MS/MS System/UltiMate 3000 HPLC. Synthetic methods and structural characterization data of the active compounds and their intermediates are described below. The 1H-NMRs and 13C-NMRs obtained for the compounds are shown as Supplementary Figures S1–S12 in the supplementary material.

## General procedure and NMR data

### Synthesis of 3,5-dinitrophthalic acid (2)

Potassium dichromate (65.014, 0.222 mol) was added slowly (one spatula for 2–3 min) to a stirring solution of 2-methyl-3,5-dinitrobenzoic acid (Lim et al., 2014) (25 g, 0.111 mol) in 200 mL of sulfuric acid at 90°C. During the addition, the temperature was maintained at <90°C. After completion of the addition, the resulting reaction mixture was stirred at 90°C for 48 h. Upon completion, the reaction mixture was cooled to room temperature, poured into the ice + water mixture, and extracted with diethyl ether. Combined organic layers were washed with water (one time) and dried over sodium sulfate. The solvent was removed under reduced pressure to obtain a slightly yellowish solid that contained both product and unreacted starting material. The mixture was refluxed with 100 mL of benzene for 15 min and decanted while hot to obtain the product in 58% yield. Usually, 7–10 washes were required.

### Synthesis of 4,6-dinitroisoindoline-1,3-dione (3)

Urea (1.20 g, 20 mmol) was added to a stirring solution of 3,5-dinitrophthalic acid (Sobral-Leite et al., 2019) (2.56 g, 10.0 mmol) in 20 mL of acetic acid. The resulting solution was stirred under refluxing conditions for 4 h. The reaction mixture was cooled to room temperature, poured into a beaker containing the ice, and stirred for a while. The obtained precipitate was filtered off, washed with water, and dried under vacuum to get the product 4,6-dinitroisoindoline-1,3-dione (Laplante and Sabatini, 2012) in 76% yield.

### Synthesis of 4,6-diaminoisoindoline-1,3-dione (4)

The dinitro compound **3** was dissolved in the required amount of methanol, transferred to a hydrogenation flask, and a five-weight percent of Pd/C was added to it. The flask was mounted on the Parr shaker instrument and shaken for 70 min at 40 Psi pressure of H_2_. After 70 min, the reaction mixture was filtered through celite. The obtained solution was transferred to a round bottom flask, and the solvent was removed using a rotavapor to get the pure product as a dirty green solid at over 90% yield.

### General procedure for the synthesis of 5-substituted amido phthalimide derivatives (**5 a–f**)

A solution of the corresponding acyl halide (0.5 mmol) in 5 mL of acetonitrile was added dropwise to a stirred solution of 4,6-diaminoisoindoline-1,3-dione **4** (Stemke-Hale et al., 2008) (0.089 g, 0.5 mmol) in 3.0 mL of N-methyl-2-pyrrollidone at 0°C and stirred at 0°C. The reaction was monitored by TLC. After completion, the reaction mixture was poured onto ice, resulting in precipitate formation. The precipitate was filtered off, washed with water several times, and dried under vacuum to get the coupled product **5** (**a–q**).

### N-(6-amino-1,3-dioxoisoindolin-4-yl)benzamide (**5a**)

Yield: 81.6%. H^1^-NMR: δ 10.79 (s, 1H), 10.45 (s, 1 H), 7.92 (d, 2 H), 7.59–7.50 (m, 7H), 7.34 (s, 1 H), 6.40(b, 2 H) (Supplementary Figure S1). C^13^-NMR: δ 170.9, 169.7, 166.5, 147.6, 145.7, 134.9, 132.3, 128.9, 128.3, 110.2, 106.2, 106.4, 104.23 (Supplementary Figure S2). HRMS: (M + Na^+^) Calculated 304.0693, observed 304.0685.

### N-(7-amino-1,3-dioxoisoindolin-5-yl)-2-phenylacetamide (**5b**)

Yield: 80.4%. H^1^NMR: δ 10.76 (bs, 1H), 10.41 (bs, 1H), 7.32 (m, 3H), 7.28 (d, 1H, J-1.2 Hz), 7.20 (m, 2H), 7.16 (d, 1H, J = 1.2 Hz), 6.37 (bs, 2H), 3.66 (s, 2H) (Supplementary Figure S3). C^13^NMR: δ 170.98, 170.23, 169.47, 147.55, 145.45, 136.01, 135.12, 129.80, 128.85, 127.15, 108.93, 106.46, 103.05, 43.65 (Supplementary Figure S4). HRMS (M + H)^+^ Calculated 234.0879, observed 234.0876.

### N-(7-amino-1,3-dioxoisoindolin-5-yl)-2-(4-(trifluoromethyl)phenyl)acetamide (**5c**)

Yield: %. H^1^NMR: δ 10.76 (bs, 1H), 10.47 (bs, 1H), 7.70 (d, 2H, J = 6.15 Hz), 7.55 (d, 2H, J = 6.15 Hz), 7.27 (d, 1H, J = 0.96 Hz), 7.16 (d, 1H, J = 0.96 Hz), 6.37 (bs, 2H), 3.80 (s, 2H) (Supplementary Figure S5). C^13^NMR: δ 170.86, 169.55, 147.72, 145.54, 141.03, 135.35, 131.13, 130.48, 125.67, 109.23, 106.46, 103.04, 43.44 (Supplementary Figure S6). HRMS (M + H)^+^ Calculated 364.0909, observed 364.0904.

### N-(7-amino-1,3-dioxoisoindolin-5-yl)-2-(3-(trifluoromethyl)phenyl)acetamide (**5d**)

Yield: %. H^1^NMR: δ 10.77 (bs, 1H), 10.48 (bs, 1H), 7.57–7.69 (m, 4H), 7.29 (d, 1H, J = 1.68 Hz), 7.15 (d, 1H, J = 1.68 Hz), 6.36 (bs, 2H), 3.81 (s, 2H) (Supplementary Figure S7). C^13^NMR: δ 171.86, 169.69, 147.72, 145.54, 137.39, 135.21, 134.04, 129.96, 123.42, 109.15, 106.39, 103.19, 43.08 (Supplementary Figure S8). HRMS (M + H)^+^ Calculated 364.0909 observed 364.0909.

### N-(7-amino-1,3-dioxoisoindolin-5-yl)adamantane-1-carboxamide (**5e**)

Yield: 86.1%. H^1^NMR: δ 10.74 (bs, 1H), 9.34 (bs, 1H), 7.40 (d, 1H, J = 1.3 Hz), 7.23 (d, 1H, J = 1.3 Hz), 6.25 (bs, 2H), 1.80 (m, 15H) (Supplementary Figure S9). C^13^NMR: δ 177.33, 171.22, 169.97, 147.75, 146.18, 110.49, 106.11, 104.55, 42.17, 41.97, 41.77, 41.55, 41.34, 38.49, 36.31, 28.02 (Supplementary Figure S10).

### N-(7-amino-1,3-dioxoisoindolin-5-yl)-2-(4-bromophenyl)acetamide (**5f**)

Yield: %. H^1^NMR: δ 10.76, (bs, 1H), 10.43 (bs, 1H), 7.52 (DD, 2H, J = 4.86 Hz and 1.71 Hz), 7.28 (m, 3H), 7.14 (d, 1H, J = 1.14 Hz), 6.36 (bs, 2H), 3.65 (s, 2H) (Supplementary Figure S11). C^13^NMR: δ 171.10, 169.89, 147.87, 145.85, 145.86, 135.51, 131.89, 120.34, 109.32, 106.50, 103.41, 43.11 (Supplementary Figure S12). HRMS (M + H)^+^ Br^79^- Calculated 374.0140, observed 374.0140; Br^81^‐ Calculated 376.0120, observed 376.0119.

### Database of derivatives of compound **4**

A database of 4-amido derivatives, 6-amido derivatives, and 4,6-diamido derivatives was created using variations in the amide carbonyl chain. The variations included aromatic/heterocyclic rings with ortho, meta, and/or para substitutions with various functional groups (-OH, -NHR, -NH2, -halogens, -Oalkyl, -ketoalkyl, or -carboxyl) in the form of esters/amides. The molecules were individually built using the Builder module in the Molecular Operating Environment (MOE) program. Initial geometric optimizations of the ligands were carried out using the standard MMFF94 force field, with a 0.001 kcal mol^−1^ energy gradient convergence criterion and a distance-dependent dielectric constant employing Gasteiger and Marsili charges. Additional geometric optimizations were performed using the semi-empirical method molecular orbital package (MOPAC). The molecules were populated into an empty database. The database was subjected to a protonation and tautomerization wash, assigned partial charges (MMFF94), and energy minimized. MOE’s Pharmacophore Search and Dock applications were used to automatically generate conformations. The generated database consisted of 344 entries used for initial docking studies. Based on the commercial availability of the acids/acid halides and the docking results, the synthesis of 4-amido derivatives, 6-amido derivatives, and 4,6-diamido derivatives was pursued. The 6-amido derivative, compound **5a,** which exhibited p70S6K1 inhibition with a potency similar to that of compound **4**, was taken as the lead compound.

### Docking studies

Docking studies were performed using the Molecular Operating Environment (MOE) program (Chemical Computing Group, Montreal, Canada). The coordinates of the protein p70S6K1 (4L3J.pdb and 3WE4.pdb) (Wang et al., 2013; Niwa et al., 2014) were taken from the Protein Data Bank (http://www.rcsb.org). Solvent molecules were removed, and hydrogen atoms were added to the template proteins using the Amber ff99 force field. Molecules were built using the Builder module in MOE, and initial geometric optimizations of the ligands were carried out using the standard MMFF94 force field, with a 0.001 kcal mol^−1^ energy gradient convergence criterion and a distance-dependent dielectric constant employing Gasteiger and Marsili charges. Additional geometric optimizations were performed using the semi-empirical method molecular orbital package (MOPAC). Docking studies were performed using the Dock module of the MOE software. The Alpha Triangle matcher placement method was used to place ligands in the binding pocket. Poses were generated by aligning ligand triplets of atoms on triplets of alpha spheres in a more systematic way than in the Alpha Triangle method. The London dG scoring function that estimates the free energy of binding of the ligand from a given pose was initially used. A rescoring of the binding pose was done using the GBVI/WSA ΔG, which is a forcefield-based scoring function that estimates the free energy of binding of the ligand from a given pose. This rescoring was initially performed with rigid receptor constraints followed by an induced fit mode where the side chains of the protein residues were free to move. The output had the top five poses with the top ‘S’ scores (the final score). The results were evaluated through visual inspection of the docked complexes. The criterion for visual inspection was that the ligand should reside in the ATP-binding pocket of the protein, close to the hinge region. The ligand pose that satisfied the initial visual inspection with the lowest “S” score was taken as the final binding pose.

### Kinase assays: information from ThermoFisher–SelectScreen services

The high-throughput and dose-response curve assays against kinases were performed using the services of SelectScreen™ Biochemical Kinase Profiling Service by Thermo Fisher Scientific (Table 2; Supplementary Figure S13). The Z ´-LYTE biochemical assay employs a fluorescence-based, coupled-enzyme format based on the differential sensitivity of phosphorylated and non-phosphorylated peptides to proteolytic cleavage. The peptide substrate is labeled with two fluorophores—one at each end—that make up a fluorescence resonance energy transfer (FRET) pair. In the primary reaction, the kinase transfers the gamma-phosphate of ATP to a single tyrosine, serine, or threonine residue in a synthetic FRET-peptide. In the secondary reaction, a site-specific protease recognizes and cleaves non-phosphorylated FRET peptides. Phosphorylation of FRET-peptides suppresses cleavage by the development reagent. Cleavage disrupts FRET between the donor (i.e., coumarin) and acceptor (i.e., fluorescein) fluorophores on the FRET-peptide, whereas uncleaved, phosphorylated FRET-peptides maintain FRET. A ratiometric method, which calculates the emission ratio of donor emission to acceptor emission after excitation of the donor fluorophore at 400 nm, is used to quantitate reaction progress, as shown in the equation below.”

$$\text{Emission ratio}=\frac{Coumarin\;emission\;\left(445\;nm\right)}{Flourescein\;emission\;\left(520\;nm\right)}.$$

### Z′-LYTE assay conditions

#### Test compounds

The test compounds were screened in 1% DMSO (final) in the well. For 10-point titrations, 3-fold serial dilutions were conducted from the starting concentration of 25 μM.

#### Peptide/kinase mixtures

All peptide/kinase mixtures are diluted to a 2× working concentration in the appropriate kinase Buffer. The 2X RPS6KB1 (p70S6K)/Ser/Thr 07 mixture is prepared in 50 mM HEPES pH 7.5, 0.01% BRIJ-35, 10 mM MgCl_2_, 1 mM EGTA. The final 10 µL kinase reaction consists of 2.87–17.7 ng RPS6KB1 (p70S6K) and 2 µM Ser/Thr 07 in 50 mM HEPES pH 7.5, 0.01% BRIJ-35, 10 mM MgCl_2_, 1 mM EGTA. After the 1 h kinase reaction incubation, 5 µL of a 1:45000 dilution of Development Reagent A is added.

#### ATP solution

All ATP Solutions are diluted to a 4X working concentration in kinase buffer (50 mM HEPES pH 7.5, 0.01% BRIJ-35, 10 mM MgCl_2_, 1 mM EGTA). ATP Km apparent was previously determined using a Z′-LYTE assay.

#### Development reagent solution

The Development Reagent was diluted in Development Buffer.

#### 10X novel protein kinase C (PKC) lipid mix

2 mg/mL phosphatidyl serine, 0.2 mg/mL DAG in 20 mM HEPES, pH 7.4, 0.3% CHAPS.

#### For the 5 mL 10X novel PKC lipid mix

1. In a glass tube, 10 mg phosphatidyl serine (Avanti Polar Lipids Part# 8400032C or 840039C) and 1 mg DAG (Avanti Polar Lipids Part# 800811C) were added.
2. The chloroform was removed from the lipid mixture by evaporating to a clear, thin film under a stream of nitrogen. The tube was continuously rotated at an angle to ensure the maximum surface area of the lipid solution and promote the thinnest film.
3. To the dried lipid mix, 5 mL resuspension buffer, 20 mM HEPES, 0.3% CHAPS, pH 7.4, was added.
4. The mixture was heated gently to 50–60°C for 1–2 min and vortexed in short intervals until the lipids were dissolved to a clear or slightly hazy solution. The lipids were typically in solution after 2–3 heat/vortex cycles.
5. The mixture was cooled to room temperature, aliquoted into single-use volumes, and stored at −20°C.

#### Z’-LYTE assay protocol

Bar-coded Corning, low volume NBS, black 384-well plate (Corning Cat. #4514).

1. 100 nL 100X test compound in 100% DMSO
2. 2.4 µL kinase buffer
3. 5 μL 2X peptide/kinase mixture
4. 2.5 µL 4X ATP solution
5. 30-s plate shake
6. 60-min kinase reaction incubation at room temperature
7. 5 μL Development Reagent solution
8. 30-s plate shake
9. 60-min Development Reaction incubation at room temperature
10. Read on the fluorescence plate reader and analyze the data

#### Cell proliferation assay

Human breast tumor cells MCF-7 subclone E3 were routinely maintained in DMEM medium (Mediatech) supplemented with non-essential amino acid (Gibco), sodium pyruvate (Gibco), L-glutamine (Gibco), gentamicin (Gibco), and 10% fetal bovine serum (FBS) (Hyclone). For the proliferation experiment, the MCF-7 monolayer was dispersed into suspension in DMEM medium with 10% FBS, and 5 × 10^5^ cells were seeded into a T-25 flask. The medium was aspirated from the cells, washed with phosphate buffer saline, and cultured with DMEM medium containing dextran-coated charcoal (DCC) FBS for 6 days. After 6 days, the cells were dispersed into suspension, counted, and 1.8 × 10^4^ cells per well in a 100 µL DCC medium was subcultured into 96-well plates. After 24 h incubation, the medium was aspirated and replaced with the test compound at 10^−5^ M. The cells were dosed again after 3 days and grown for another 3 days. Measurement for cell proliferation was performed with alamarBlue (Bio-Rad) on a plate reader (BioTek Synergy Neo2) with 560 nm excitation and 590 nm emission. The experiments were performed in quadruplicate.

#### Western blotting

Total proteins were isolated using standard protocols as previously described (Greenberg et al., 2022; Kim H. et al., 2013). In brief, ice-cold RIPA lysis buffer containing phosphatase and protease inhibitors (Santa Cruz Biotechnology, Dallas, TX, USA) was used to lyse cells. The clarified lysate protein concentration was measured using Bradford reagent (Thermo Scientific, Waltham, MA, USA). The proteins were separated using a 4%–20% SDS-PAGE gradient gel and transferred onto a PVDF membrane (Bio-Rad, Hercules, CA, USA). A 1X blocking buffer was used to block the non-specific binding sites. The membranes were washed with Tris-buffered saline (Bio-Rad) containing 0.1% Tween-20 (Sigma-Aldrich, St. Louis, MO, USA). Membranes were incubated overnight with primary antibodies at 4°C. Protein extracts were subjected to immunoblot analysis using antibodies against S6 Ribosomal Protein (5G10) (Cell Signaling Technology, Danvers, MA, USA), Phospho-S6 Ribosomal Protein (Ser240/244) (Cell Signaling Technology), Phospho-S6 Ribosomal Protein (Ser235/236) (D57.2.2E) (Cell Signaling Technology), or GAPDH (Santa Cruz Biotechnology, Dallas, TX). Immune complexes were detected with appropriate secondary antibodies from Invitrogen (Camarillo, CA, USA) and Clarity Western ECL Substrate (Bio-Rad), as described (Greenberg et al., 2022). Immunoblot signals were captured using the Bio-Rad Imager Bio-Rad ChemiDoc Touch Imaging System (Bio-Rad) system (Supplementary Figures S14, S15). Immune band densitometry was performed using ImageJ Software (NIH, Bethesda, MD, USA, http://imagej.nih.gov/ij/accessed on 4 November 2021). Results were expressed as the standard error of the mean (±SEM). Significant changes from controls or E2 treated group were determined by a two-tailed Student’s t-test, and *p*-values of <0.05 were considered significant.

## Results and discussion

**FIGURE 2:**
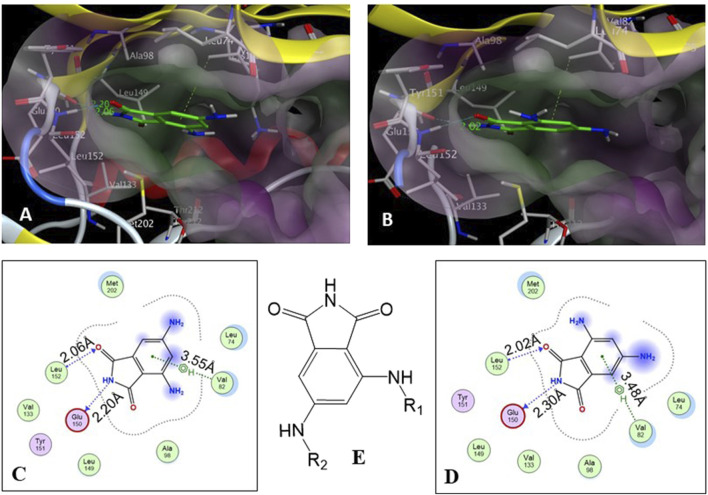
**(A, B)** Binding modes of core structure **4** from a docking study in the ATP-binding pocket of the p70S6K1 crystal structure (4L3J.pdb). The surface map of the binding cavity is colored by lipophilicity (pink—hydrophilic, green—lipophilic). Molecule 4 is shown as a stick model, with the protein depicted as a ribbon model. **(C)** Ligand interaction picture for the binding mode shown in **(A)**. **(D)** Ligand interaction picture for the binding mode shown in **(B)**. **(E)** Two points of derivatization of the core structure with substituents R_1_ and R_2_.

The core structure of 4,6-diaminoisoindolin-1,3-dione (compound **4** in Figure 1) was designed based on the natural product staurosporine. The core structure has multiple points of derivatization, with the aniline nitrogens providing straightforward points for the attachment of new structural extensions. We evaluated the docking of compound **4** on the p70S6K1 3D crystal structure (Figure 2, PDB ID 4L3J), which showed two preferred binding modes (Figures 2A, B) for the molecule. Both binding modes have the amide ring of **4**, making two hydrogen bonds with the hinge region backbone of Glu150 and Leu152 (PDB ID 4L3J) and an aromatic π-H interaction with the side chain of Val82 (PDB ID 4L3J). Based on the orientation of the molecule against the hinge region (Figures 2C, D), the aniline groups orient toward the different areas of the ATP-binding pocket. In binding mode 1 (Figures 2A, C), the 4-amino group is reaching out to the site bound by the Lys residue on b3, and the C-helix and 6-amino group is pointing toward the A-loop that forms the critical substrate binding site. In binding mode 2 (Figures 2B, D), the 4-amino group is oriented toward the C-terminal region of the kinase, and the 6-amino group extends to the A-loop. Depending on the substituents on the 4- and 6-amino groups (Figure 2E), one of the two binding modes would be preferred.

**FIGURE 3:**
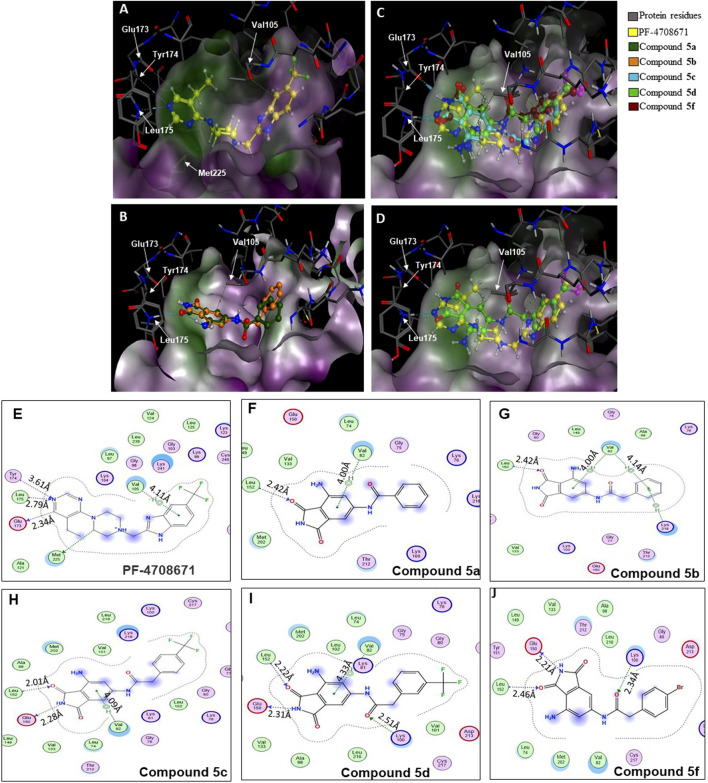
Binding modes of **(A)** PF-4708671 (3WE4.pdb), **(B)** overlay of docked compounds **5a** and **5b**, and **(C)** overlay of PF-4708671 and docked compounds **5c**, **5d**, and **5f**. **(D)** The overlay of PF-4708671 and docked compound **5d** is depicted from a docking study of the compounds in the ATP-binding pocket of the p70S6K1 crystal structure (4L3J.pdb). The compounds are shown as ball and stick models. The binding site residues are shown as stick models. The residues in **(A)** to **(D)** are labeled with amino acid numbers in the crystal structure 3WE4.pdb. The surface map of the binding cavity is colored by lipophilicity (pink—hydrophilic, green—lipophilic). Ligand interaction pictures of the compounds are captured in Figures **(E–J)** for PF-4708671 and compounds **5a**, **5b**, **5c**, **5d**, and **5f**, respectively.

A database of 4-amido derivatives, 6-amido derivatives, and 4,6-diamido derivatives was created (see Methods). The database was docked to the ATP-binding site of the p70S6K1 protein. The 6-amido derivative **5a** adopted binding mode 2, where the benzene ring of the 6-benzamide group occupied the cleft bound by the invariant Lys and the hydrophobic sidechains of the residues of A-loop (cleft A). The aromatic rings of p70S6K1 specific inhibitor PF-4708671 face the hinge region, making hydrogen bonds to Glu173, Tyr174, and Leu175 (PDB ID 3WE4), with hydrophobic interactions of the piperazine methylenes to Met225 (PDB ID 3WE4). The trifluoromethyl substituted aromatic ring reaches cleft A. A comparison with the binding mode of the p70S6K1-specific inhibitor PF-4708671 (Figure 3A) showed that the initial derivative **5a** of our lead compound **4** exhibited a similar binding orientation, with hydrogen bonds to the hinge region residues Glu173 and Leu175 (PDB ID 3WE4), an aromatic π–methyl interaction of the phthalimido benzene ring with Val105 (PDB ID 3WE4), and the substituent benzoyl group reaching the cleft A (Figure 3B). Recognizing the potential of improving the protein–ligand interactions by extending the reach of the phenyl moiety and incorporating a trifluouromethyl group similar to the one on PF-4708671, we designed and synthesized compounds **5b–5f** (Figures 3B–F).

**FIGURE 4:**
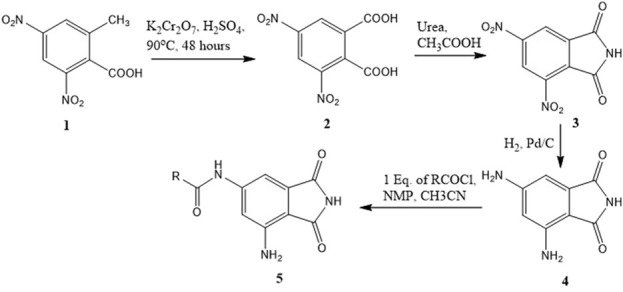
Synthetic scheme for the preparation of 6-amido-4-aminoisoindolin-1,3-dione derivatives.

A series of compounds (**5b–5f**) based on the core structure **4** were prepared using the synthetic scheme in Figure 4. Preparation of **4** from 2-methyl-3,5-dinitrobenzoic acid was accomplished using well-established reactions, as depicted in Figure 4. Oxidation of the benzylic methyl in **1** to the carboxylic acid was achieved using the Jones reaction at 90°C to form 3,5-dinitrophthalic acid **2**. Refluxing **2** with urea in acetic acid accomplished the formation of the phthalimide **3**. Reduction of nitro groups with H_2_ in the presence of Pd/C as catalysts provided **4** in quantitative yields. The acylation of **4** required careful control of the number of equivalents of the acyl chloride to prevent the formation of 4,6-diamidoisoindolin-1,3-dione. Products from all reactions were characterized by 1H-NMR and 13C-NMR. The final compounds **5a–e** were additionally characterized by LC-MSMS.

The SelectScreenTM Biochemical Kinase Profiling Service by ThermoFisher Scientific was used to perform a high-throughput kinase inhibition assay at a single concentration as well as 10-point titrations to generate the dose-response curves for IC_50_ calculations. The Z′-LYTE biochemical assay, which is a FRET-based assay, was used to analyze the compounds for p70S6K1 inhibition. Compounds **5a–f** were subjected to an initial high-throughput screening against p70S6K1 at 10 µM concentration (Table 1). Those compounds that showed ≥70% inhibition in the high-throughput assay were subjected to 10-point titrations to obtain the IC_50_ values of inhibition (Table 2). Compounds **5d** and **5f** exhibited the highest percent inhibition of p70S6K1 at 10 µM concentration, 94% and 93%. Compound **5e** had the lowest value of kinase inhibition at 22%. Although the variation in percent kinase inhibition at 10 µM concentration for the compounds **5a, 5b, 5c, 5d**, and **5f** were from 71% to 94%, the IC_50_ values of p70S6K1 inhibition showed large variations. Compounds **5b, 5c, 5d**, and **5f** produced well-defined dose-response curves, while **5a** showed huge variations in inhibition at >2.5 µM compound concentration (Supplementary Material, Supplementary Figure S13). Compound **5b,** which is extended by a methylene moiety and contains a phenylacetoyl instead of a benzoyl group, was able to penetrate deeper into the cleft A and made additional π-H interactions with the side chain branched methyl of Val82 (Figures 3B, G, PDB ID 4L3J). This improved the potency of the compound **5b** to an IC_50_ value of 0.40 µM. Analogous to the trifluoromethyl group of PF-4708671, a trifluoromethyl group was introduced in the para (**5c**) and meta (**5d**) positions, and an additional analog was synthesized with a para bromo group (**5f**) on the phenylacetamido side chain. The binding mode of **5c** showed that phenyl with the *para-*trifluoromethyl phenyl substituent was oriented in an identical manner in cleft A, with comparable positioning of the benzene ring (Figures 3C, H). However, the IC_50_ value of p70S6K1 inhibition for **5c** was 5.45 µM. The binding mode of **5d** and **5f** showed a repositioning of the benzene to accommodate the trifluouromethyl/bromo groups in cleft A (Figures 3C, D, I, J). This caused the amide carbonyl to switch positions, enabling it to form a new H-bond with the invariant Lys100 residue. The IC_50_ values of inhibition for **5d** and **5f** were remarkably higher, at 0.51 µM and 0.75 µM.

**TABLE 1:** Percent inhibition of p70S6K1 activity by the compounds at 10 µM concentration.

Compound	% Inhibition
4	70
5a	71
5b	98
5c	80
5d	94
5e	22
5f	93

**TABLE 2:** IC_50_ values of inhibition of p70S6K1 by compounds that showed ≥70% inhibition at 10 µM concentration.

Compound	IC_50_ µM
PF-4708671	0.18
4	1.20
5a	>2.78
5b	0.40
5c	5.45
5d	0.51
5f	0.75

**FIGURE 5:**
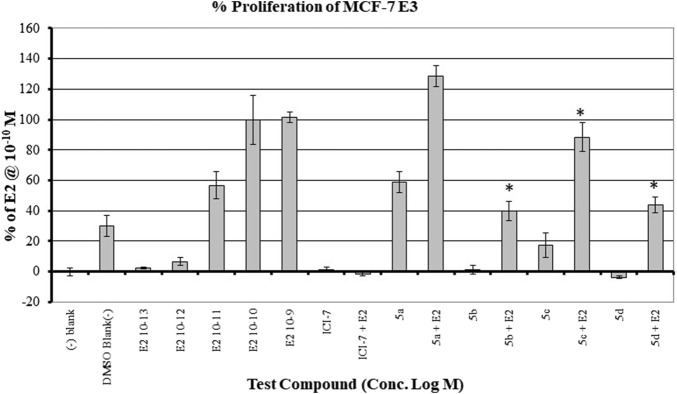
Effect of the compounds on the proliferation of MCF E3 breast cancer cells under estrogen-deprived conditions and upon the addition of E2 (estradiol). The compound concentrations were 10^−5^ M, and E2 concentrations in compound-treated cells were 10^−10^ M. The effect of increasing concentrations of E2 (10^−13^ M to 10^−9^ M) on the proliferation of MCF7 cells is shown here. All experiments were performed in quadruplicate and are shown as mean ± S.D. *p < 0.05 compared to the E_2_-treated group.

The MCF7 cell line is often used in studies involving ERα overexpressing BC. It is well-established that overexpression of estrogen leads to overexpression of p70S6K1 due to the costimulatory relationship between them, thereby driving the progression of BC (Fullwood et al., 2009; Maruani et al., 2012). The knockdown or absence of estrogen was partially abrogated by p70S6K1 rescuing and promoting ERα activity (Holz, 2012; Yamnik et al., 2009). For these reasons, MCF7 cells were used for the proliferation studies in the presence of our p70S6K1 inhibitors. MCF7 E3 cells were grown under estrogen-deprived conditions and treated with 10 μM concentrations of the compounds with and without E_2_ (10^−10^ M). The proliferation was measured using alamarBlue (Figure 5). Compound **5b**, with the best p70S6K1 inhibition potency of 0.4 μM IC_50_ value, inhibited the proliferation of MCF7 E3 cells by 60% in the presence of E2. Compounds **5c, 5d**, and **5f**, with p70S6K1 inhibition IC_50_ values of 5.45 μM, 0.51 μM, and 0.75 μM, inhibited the proliferation by 35%, 56%, and 40%, respectively. The trend of MCF7 E3 proliferation was directly proportional to the p70S6K1 inhibition potency. Interestingly, among the series of compounds assessed for effects on cell viability, **5b** and **5d** showed greater than 90% inhibition of p70S6K1 at 10 µM (Table 1), as well as the sub-µM IC_50_ values (Table 2) compared to the other compounds. This further suggests that the antineoplastic mechanism of compounds **5b** and **5d** is elicited through inhibition of p70S6K1.

**FIGURE 6:**
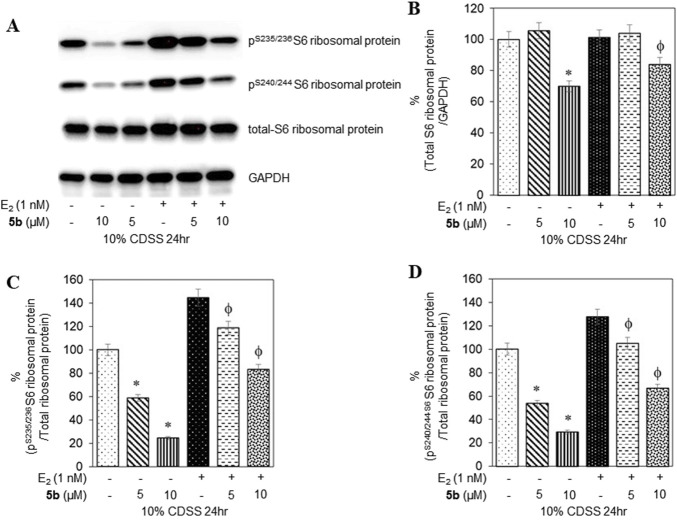
MCF7 cells deprived of estrogen were treated with 5 μM and 10 μM concentrations of compound **5b** and compound **5b + E2**. After 24 h, the cell lysates were probed with rpS6 antibody **(A, B)** and phospho rpS6 antibodies for S235/236 **(A, C)** and S240/244 **(A, D)**. The values are obtained from three independent experiments and shown as mean ± S.D. *p < 0.05 compared to the control group and ^ϕ^ *p* < 0.05 compared to the E_2_-treated group.

**FIGURE 7:**
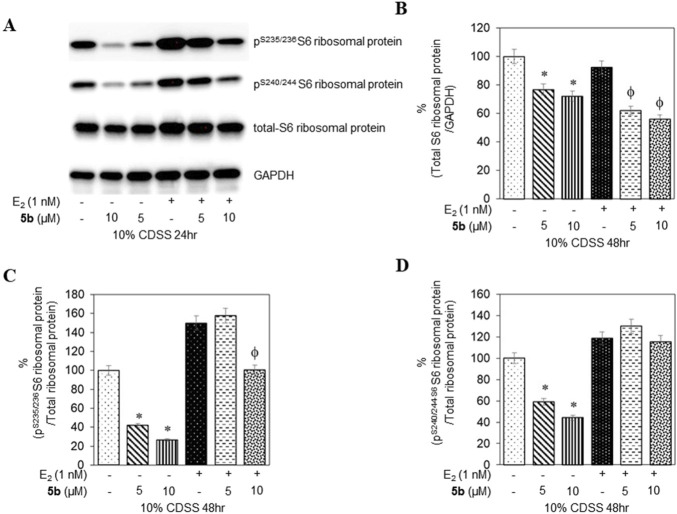
MCF7 cells deprived of estrogen were treated with 5 μM and 10 μM concentrations of compound **5b** and compound **5b + E2**. After 48 h, the cell lysates were probed with rpS6 antibody **(A, B)** and phospho rpS6 antibodies for S235/236 **(A, C)** and S240/244 **(A, D)**. The values are obtained from three independent experiments and shown as mean ± S.D. *p < 0.05 compared to the control group and ^ϕ^ *p* < 0.05 compared to the E_2_-treated group.

To confirm that the active compounds inhibited the kinase activity of the p70S6K1 protein, we performed immunoblots against the P70S6K1 substrate, S6 of 40S ribosomal subunit (rpS6). The first substrate identified for downstream phosphorylation by p70S6K1 S6 protein is phosphorylated at five residues (S235, S236, S240, S244, and S247) in sequential order by p70S6K1 (Magnuson et al., 2012; Tahir Majeed et al., 2019; Meyuhas, 2015). In brief, MCF7 cells were estrogen deprived and subsequently exposed to compound **5b** at concentrations of 5 μM and 1 nM in the presence or absence of β estradiol (Figures 6A, 7A; Supplementary Figures S14, S15). The total rpS6 protein and rpS6 phosphorylation clusters at S235/236 and 240/S244 were assessed by immunoblot at 24 h (Figure 6A; Supplementary Figure S14) and 48 h (Figure 7A; Supplementary Figure S15). Graphical representation of the band intensity for the total rpS6 protein (Figures 6B, 7B) and the phosphorylated rpS6 clusters at S235/236 (Figures 6C, 7C) and 240/S244 (Figures 6D, 7D) as a percentage of GADPH at 24 h and 48 h indicate the reduction in downstream phosphorylation of rpS6 protein in the compound **5b**-treated cells. The presence of β estradiol resulted in significant upregulation of S6 protein expression and commensurate phosphorylation. Compound **5b** exposure, however, resulted in significant decreases at both 5 µM and 10 µM. These findings provide *in vitro* evidence that compound **5b** inhibits p70S6K1 kinase activity in breast carcinoma cells. Compounds **5a–5f** have the phthalimide core structure that is frequently conjugated to pharmacophoric molecules with the goal of improving the pharmacokinetic and pharmacodynamic properties of potential therapeutics to achieve low toxic effects (Sharma et al., 2010). Compounds **5b**, **5d**, and **5f** exhibit nanomolar inhibition potency for p70S6K1 that is comparable to that of PF-4708671. Although PF-4708671 is undergoing evaluation in phase trials, other p70S6K1 inhibitors have not advanced in clinical trials due to toxicity issues.

## Conclusion

The critical role of p70S6K1 in breast cancer pathways and the correlation of its expression to the aggressiveness of the tumors render it an ideal target for the development of therapeutics. We have performed investigations of the therapeutic potential of a class of protein kinase molecules we developed (Sridhar et al., 2024). The protein–ligand interaction knowledge derived from the reported crystal structures for the protein in complex with PF-4708671 was used to increase the inhibition potency of our new compounds. The substitutions on the phenylacetyl group do not seem to improve the inhibition potency, with the unsubstituted group showing the best potency among the series. The growth inhibition evidenced in the ER + MCF7 breast cancer cells and the decrease in phosphorylation of downstream rpS6 residues confirms that targeting p70S6K1 has high therapeutic potential. Further structural modifications to reach other regions of the binding pocket to improve the p70S6K1 inhibition potency will be the future goal.

## Data Availability

The datasets presented in this study can be found in online repositories. The names of the repository/repositories and accession number(s) can be found in the article/Supplementary Material.
