# Supplementary material for: Development of 6-amido-4-aminoisoindolyn-1,3-diones as p70S6K1 inhibitors and potential breast cancer therapeutics
Source: Front Mol Biosci. 2024 Dec 19;11:1481912. doi: 10.3389/fmolb.2024.1481912 (PMC11694070; doi:10.3389/fmolb.2024.1481912)
Supplement: Supplementary file 1 [file Presentation1.pptx]

## Slide 1
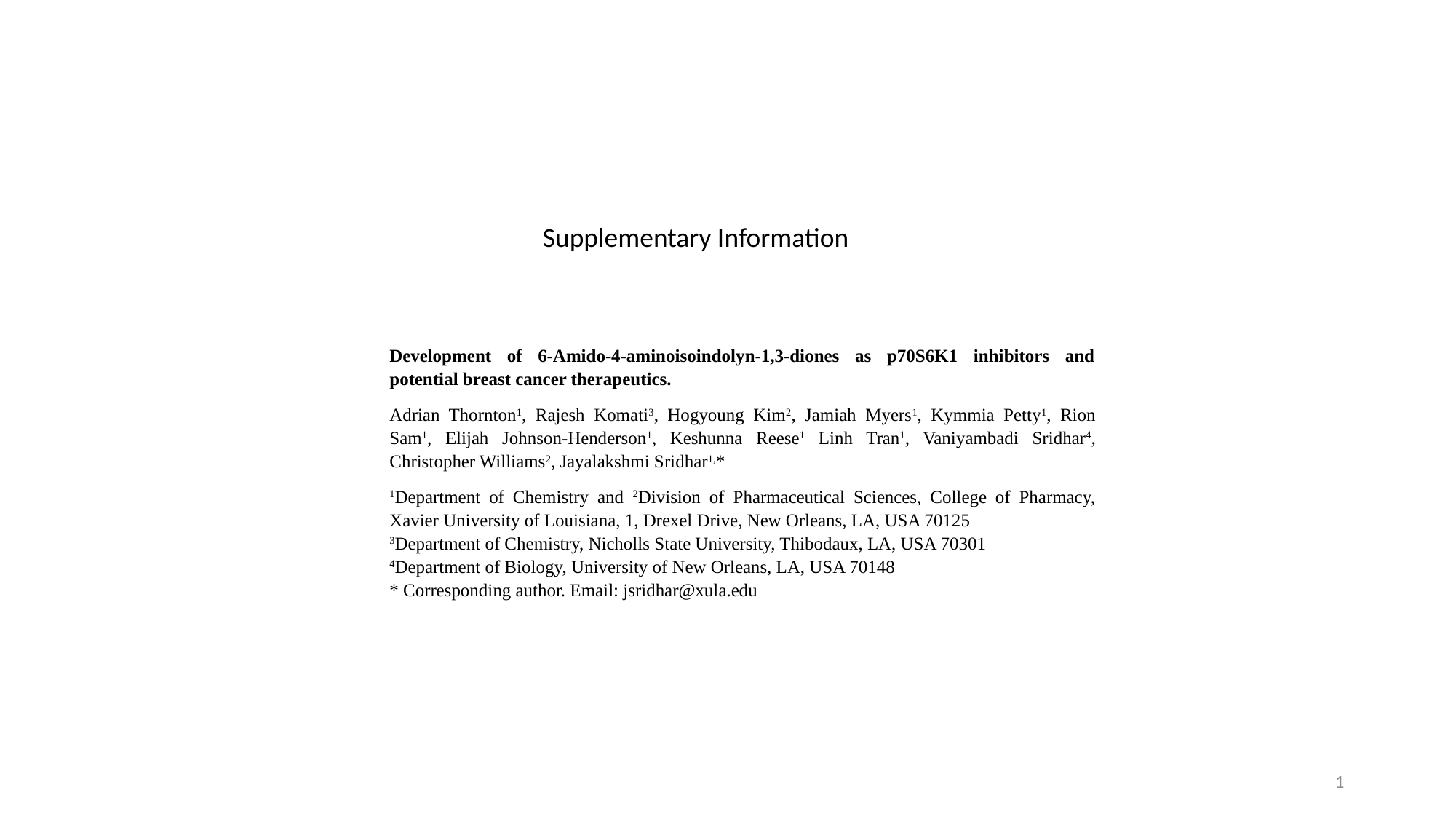

Supplementary Information
Development of 6-Amido-4-aminoisoindolyn-1,3-diones as p70S6K1 inhibitors and potential breast cancer therapeutics.
Adrian Thornton1, Rajesh Komati3, Hogyoung Kim2, Jamiah Myers1, Kymmia Petty1, Rion Sam1, Elijah Johnson-Henderson1, Keshunna Reese1 Linh Tran1, Vaniyambadi Sridhar4, Christopher Williams2, Jayalakshmi Sridhar1,*
1Department of Chemistry and 2Division of Pharmaceutical Sciences, College of Pharmacy, Xavier University of Louisiana, 1, Drexel Drive, New Orleans, LA, USA 70125
3Department of Chemistry, Nicholls State University, Thibodaux, LA, USA 70301
4Department of Biology, University of New Orleans, LA, USA 70148
* Corresponding author. Email: jsridhar@xula.edu
1

## Slide 2
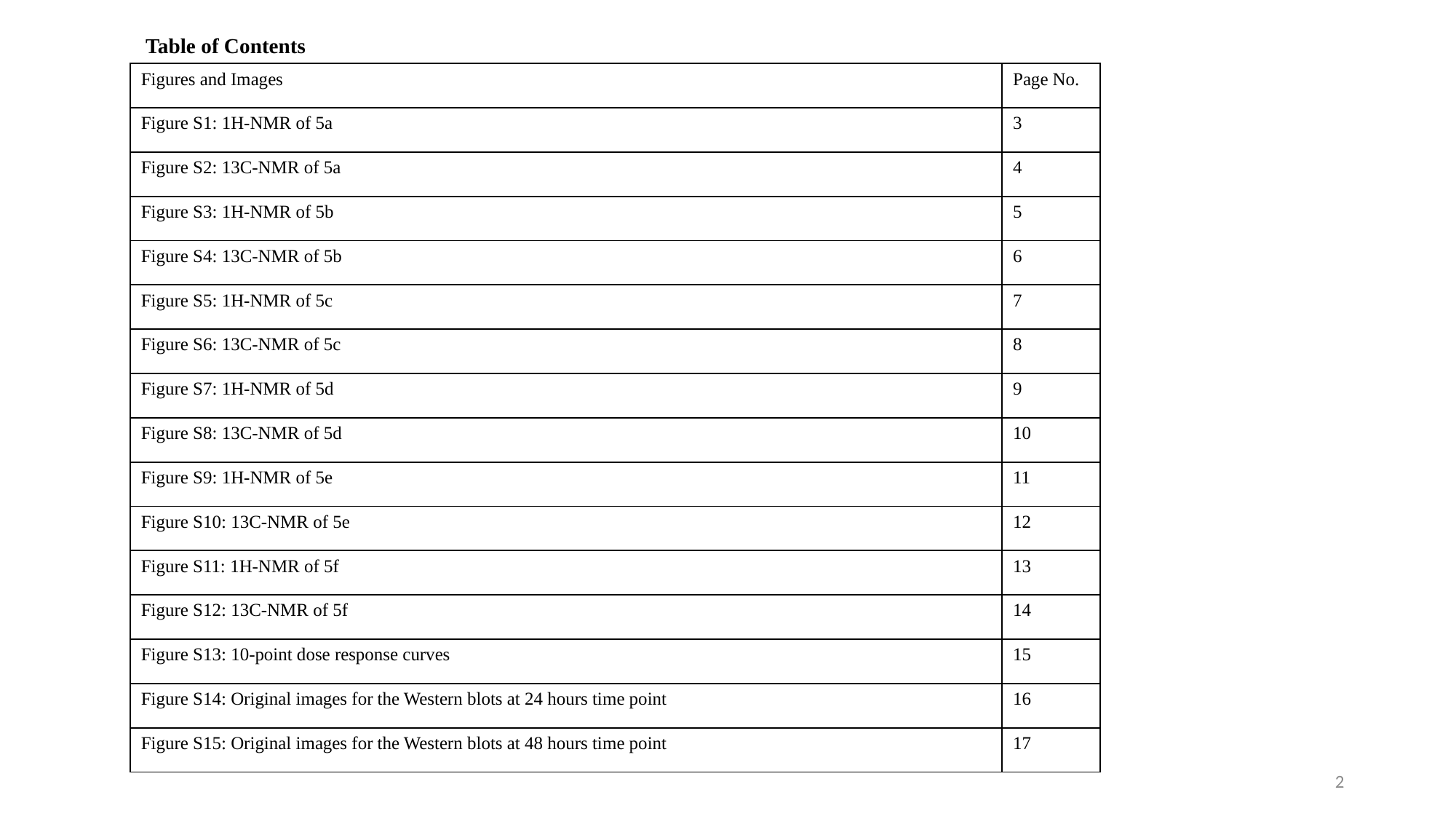

Table of Contents
| Figures and Images | Page No. |
| --- | --- |
| Figure S1: 1H-NMR of 5a | 3 |
| Figure S2: 13C-NMR of 5a | 4 |
| Figure S3: 1H-NMR of 5b | 5 |
| Figure S4: 13C-NMR of 5b | 6 |
| Figure S5: 1H-NMR of 5c | 7 |
| Figure S6: 13C-NMR of 5c | 8 |
| Figure S7: 1H-NMR of 5d | 9 |
| Figure S8: 13C-NMR of 5d | 10 |
| Figure S9: 1H-NMR of 5e | 11 |
| Figure S10: 13C-NMR of 5e | 12 |
| Figure S11: 1H-NMR of 5f | 13 |
| Figure S12: 13C-NMR of 5f | 14 |
| Figure S13: 10-point dose response curves | 15 |
| Figure S14: Original images for the Western blots at 24 hours time point | 16 |
| Figure S15: Original images for the Western blots at 48 hours time point | 17 |
2

## Slide 3
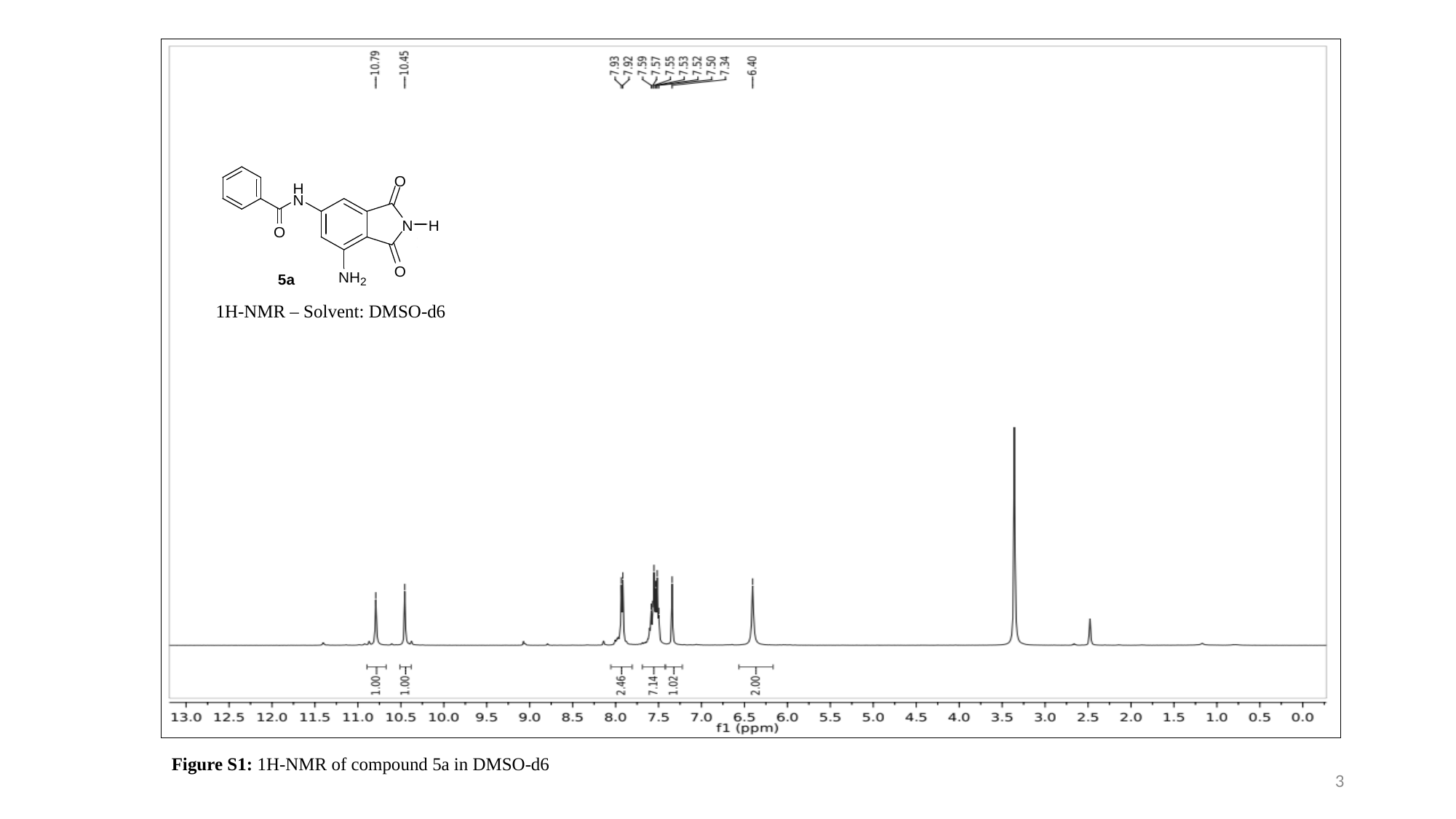

1H-NMR – Solvent: DMSO-d6
Figure S1: 1H-NMR of compound 5a in DMSO-d6
3

## Slide 4
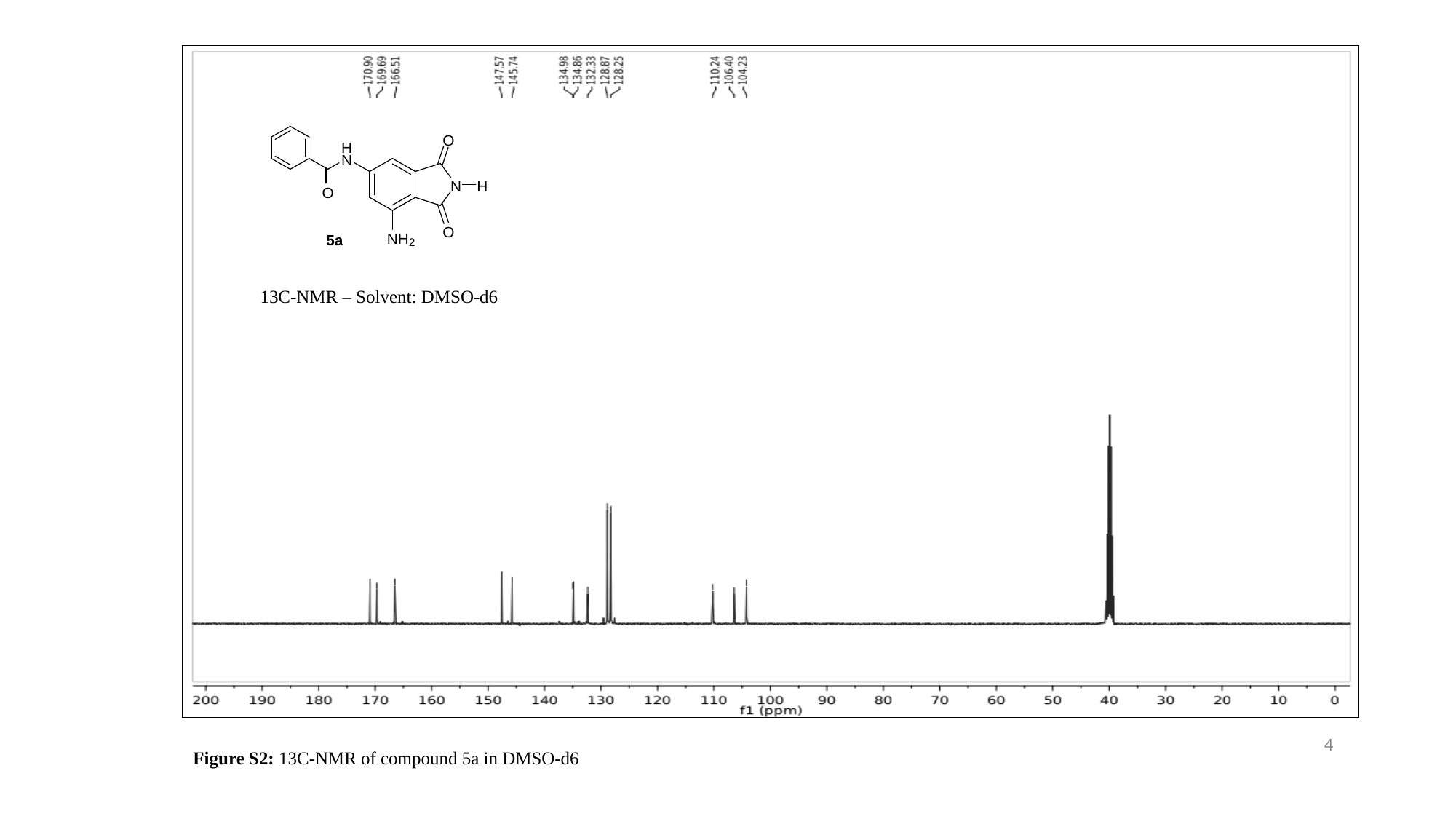

13C-NMR – Solvent: DMSO-d6
4
Figure S2: 13C-NMR of compound 5a in DMSO-d6

## Slide 5
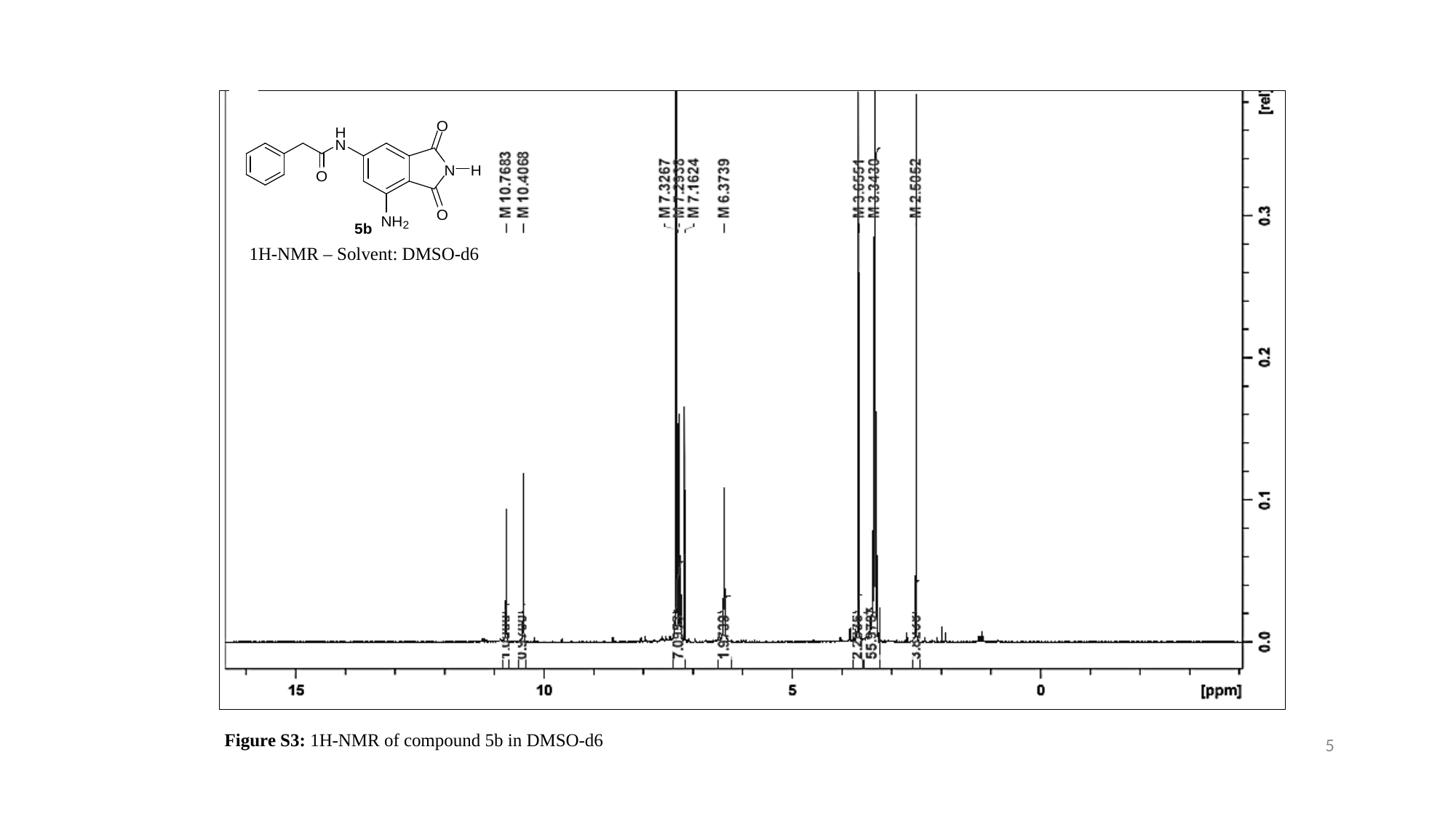

1H-NMR – Solvent: DMSO-d6
Figure S3: 1H-NMR of compound 5b in DMSO-d6
5

## Slide 6
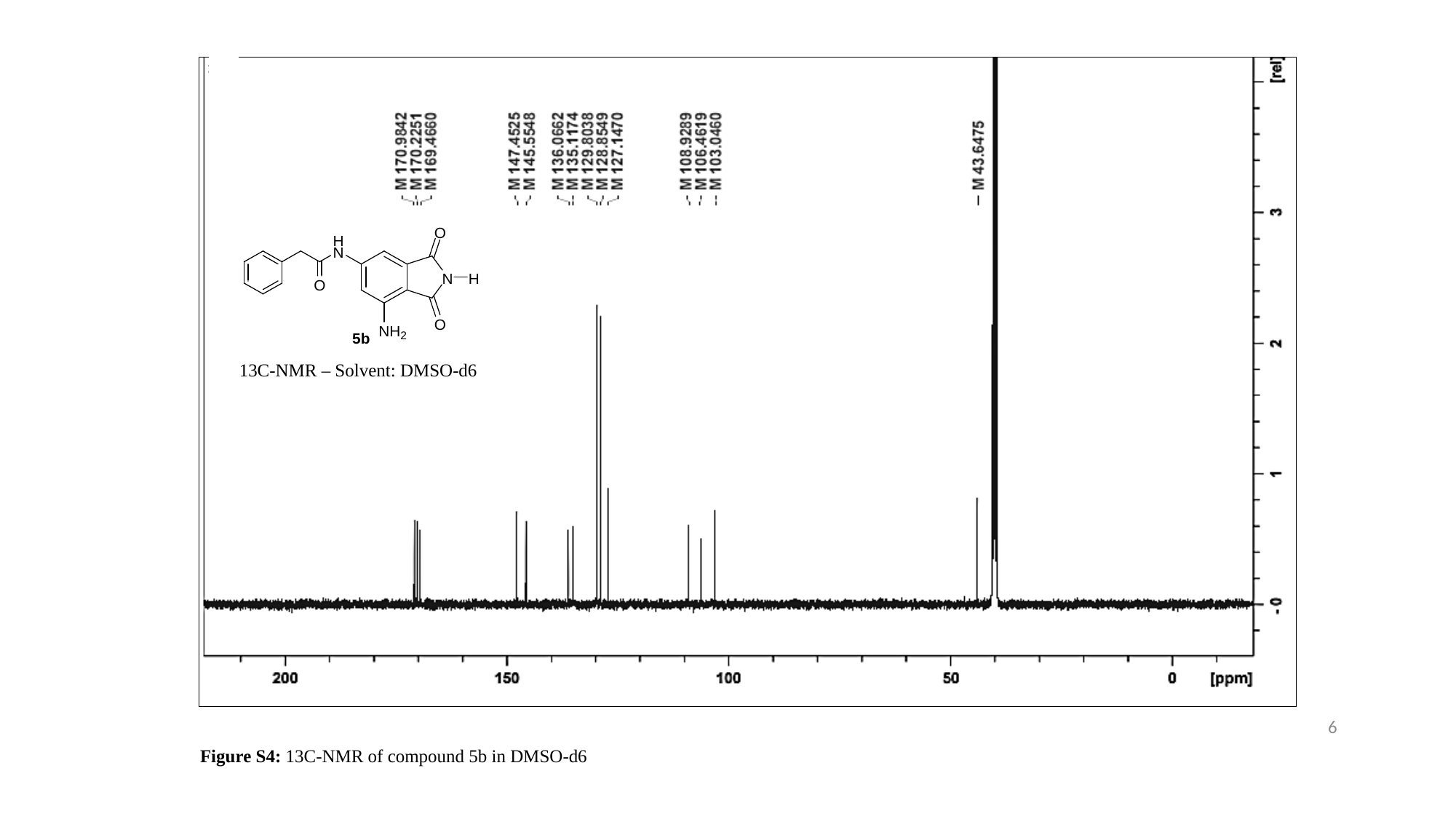

13C-NMR – Solvent: DMSO-d6
6
Figure S4: 13C-NMR of compound 5b in DMSO-d6

## Slide 7
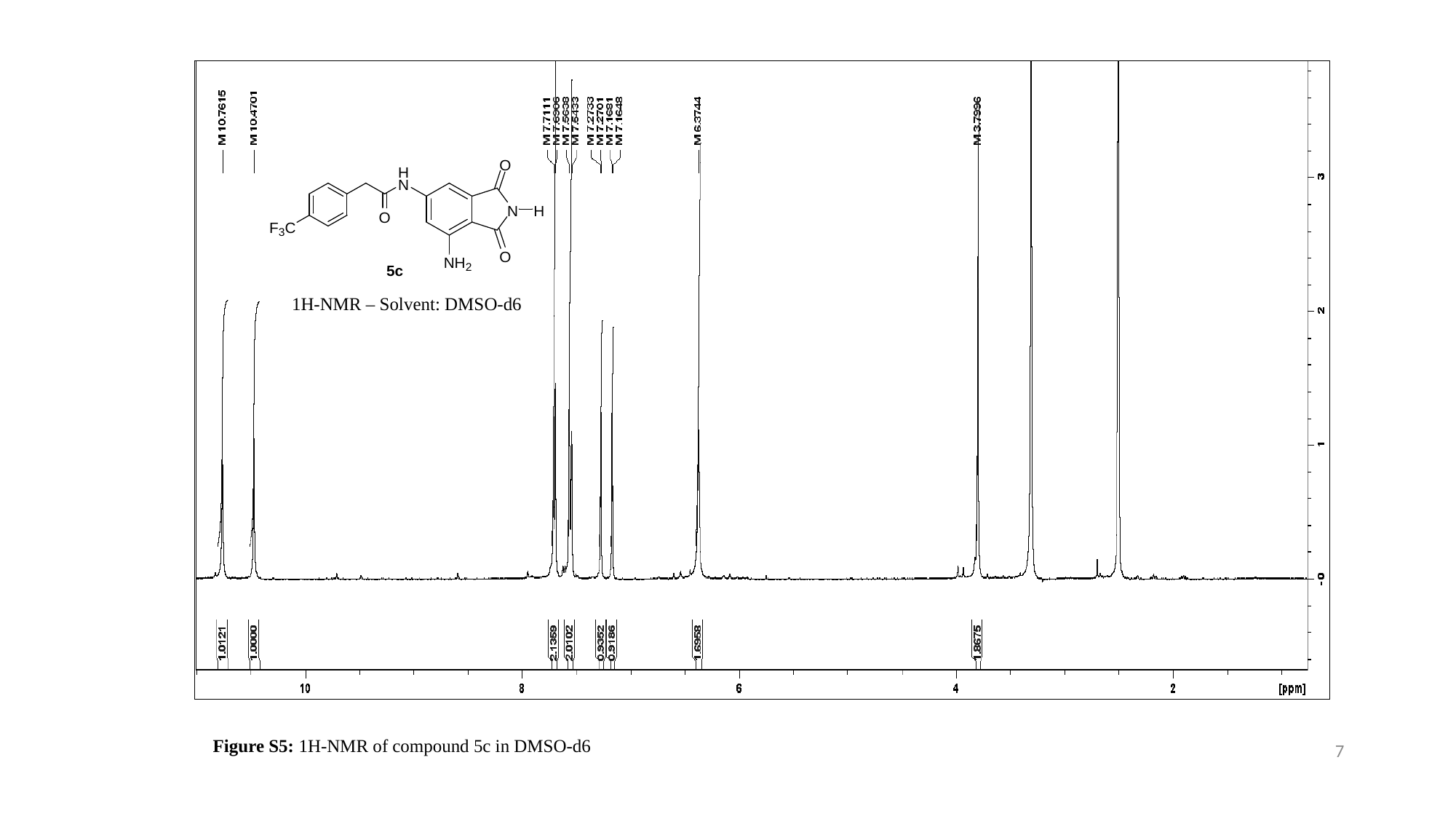

1H-NMR – Solvent: DMSO-d6
Figure S5: 1H-NMR of compound 5c in DMSO-d6
7

## Slide 8
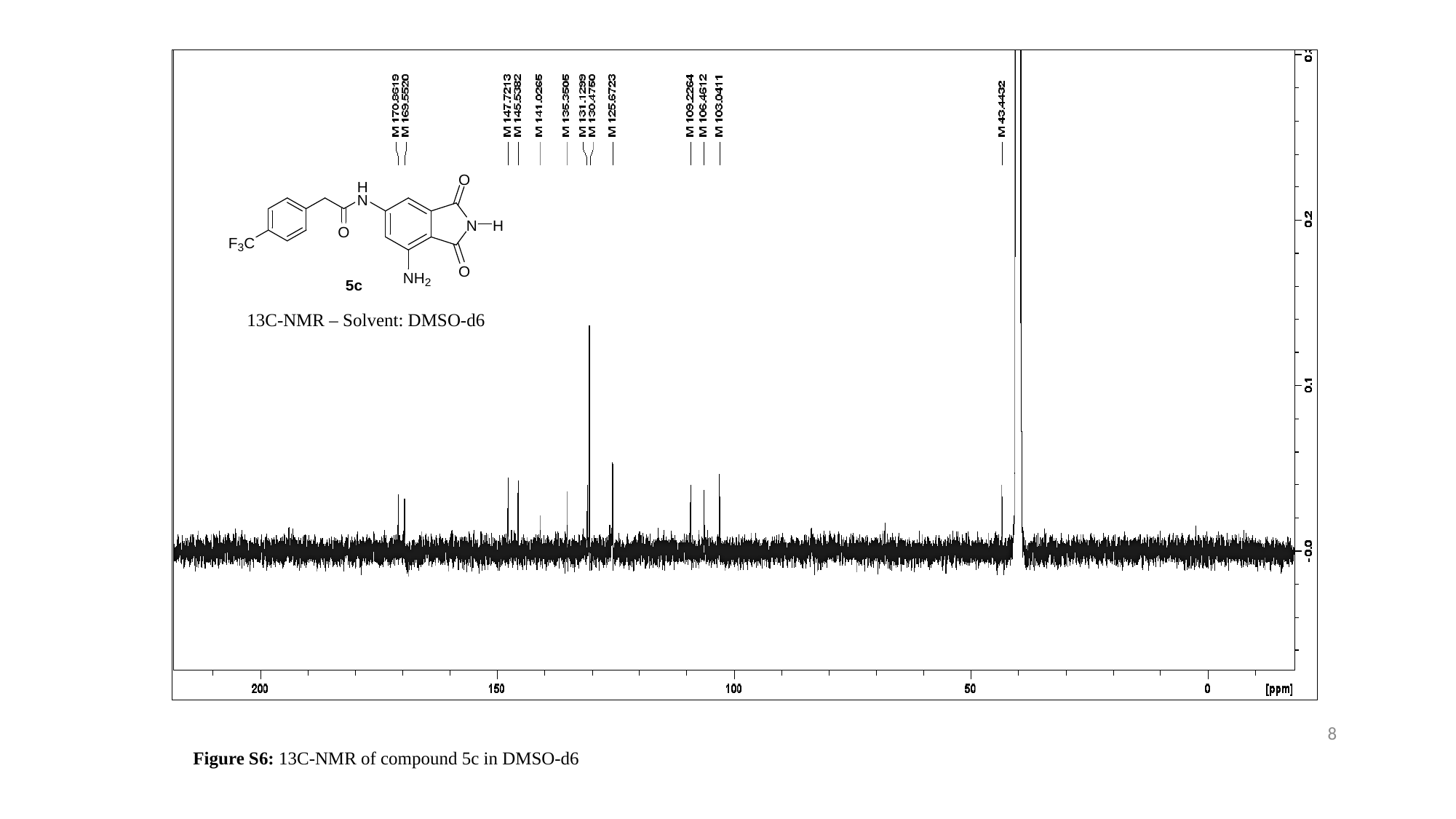

13C-NMR – Solvent: DMSO-d6
8
Figure S6: 13C-NMR of compound 5c in DMSO-d6

## Slide 9
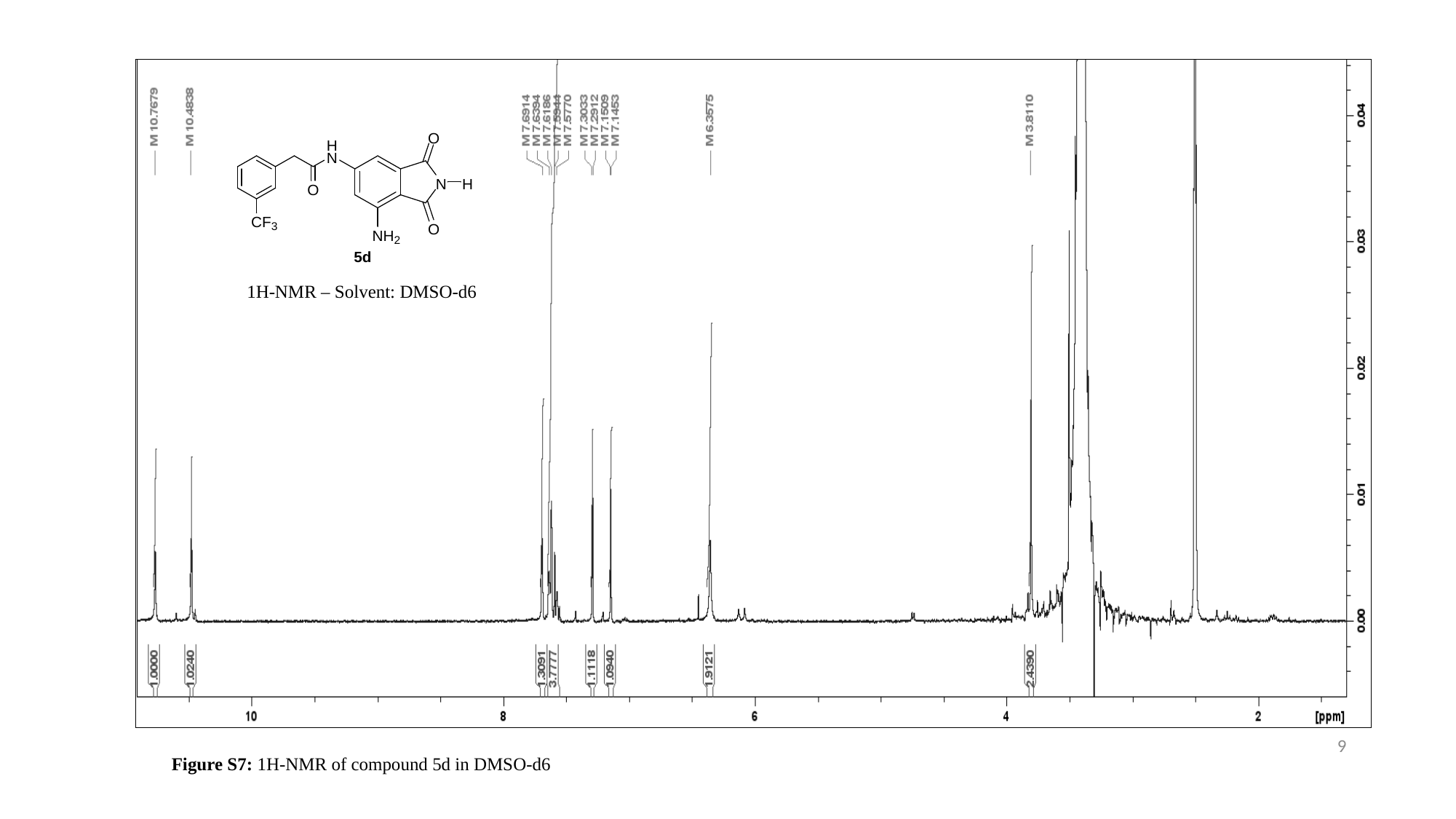

1H-NMR – Solvent: DMSO-d6
9
Figure S7: 1H-NMR of compound 5d in DMSO-d6

## Slide 10
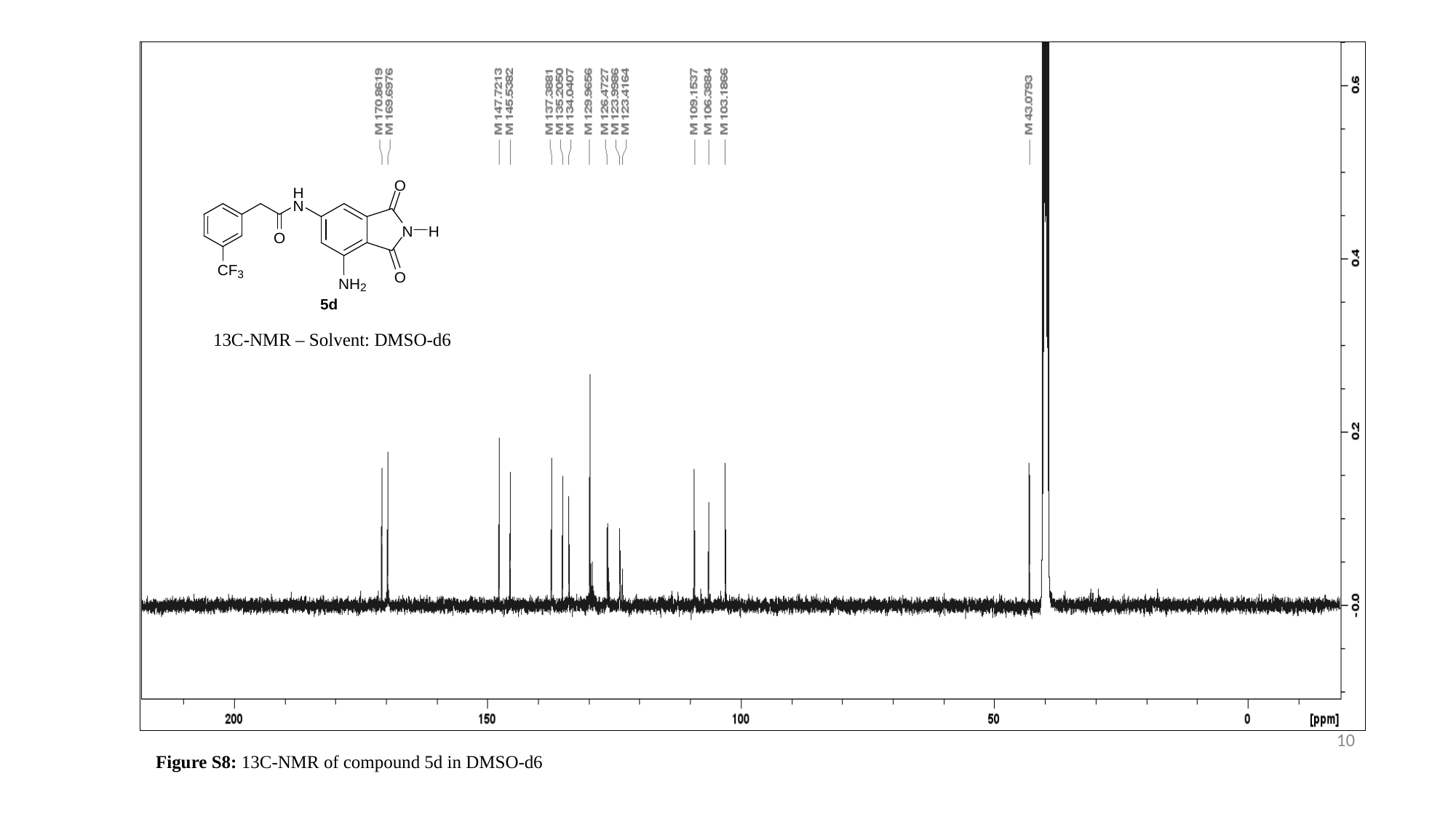

13C-NMR – Solvent: DMSO-d6
10
Figure S8: 13C-NMR of compound 5d in DMSO-d6

## Slide 11
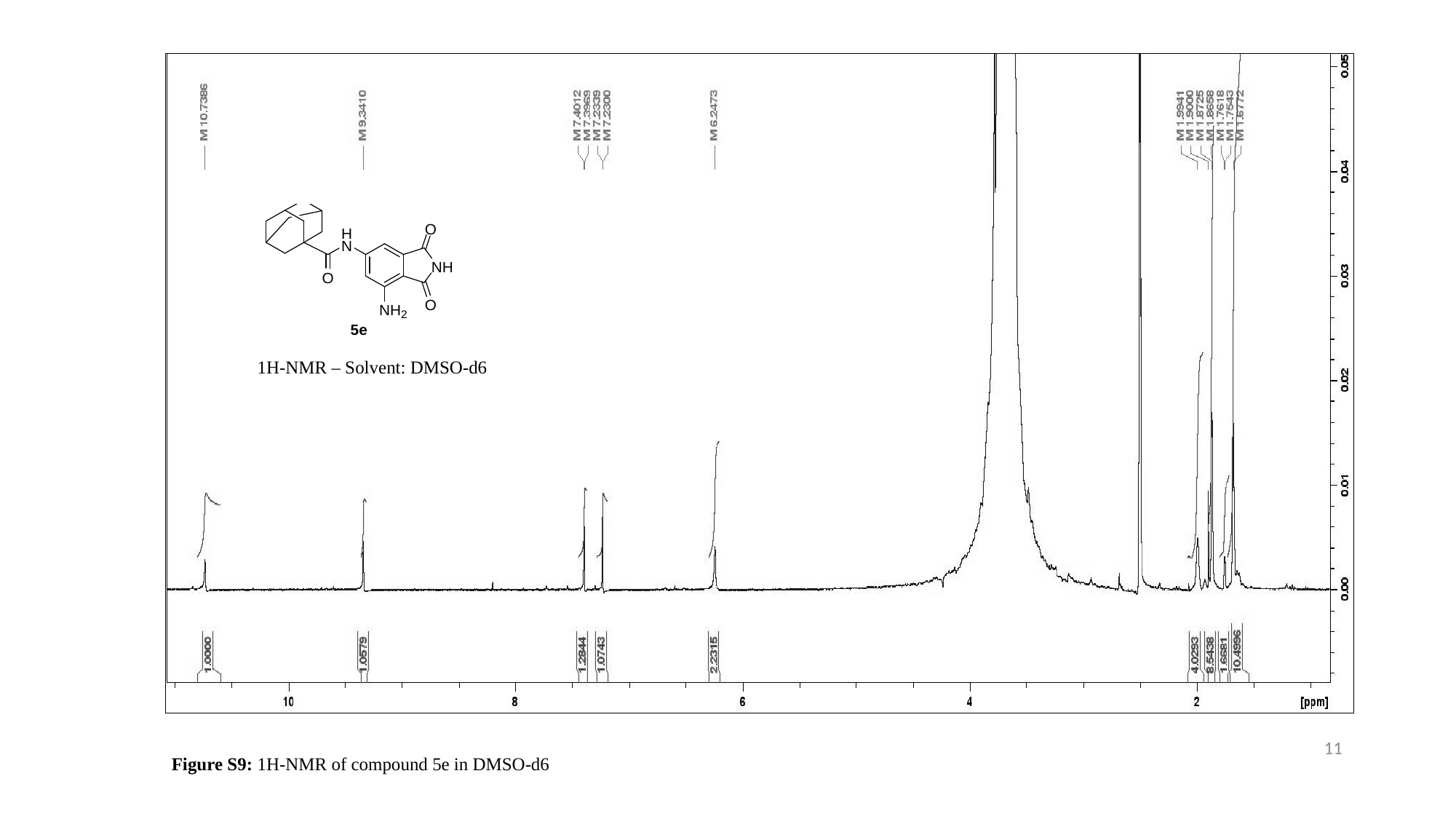

1H-NMR – Solvent: DMSO-d6
11
Figure S9: 1H-NMR of compound 5e in DMSO-d6

## Slide 12
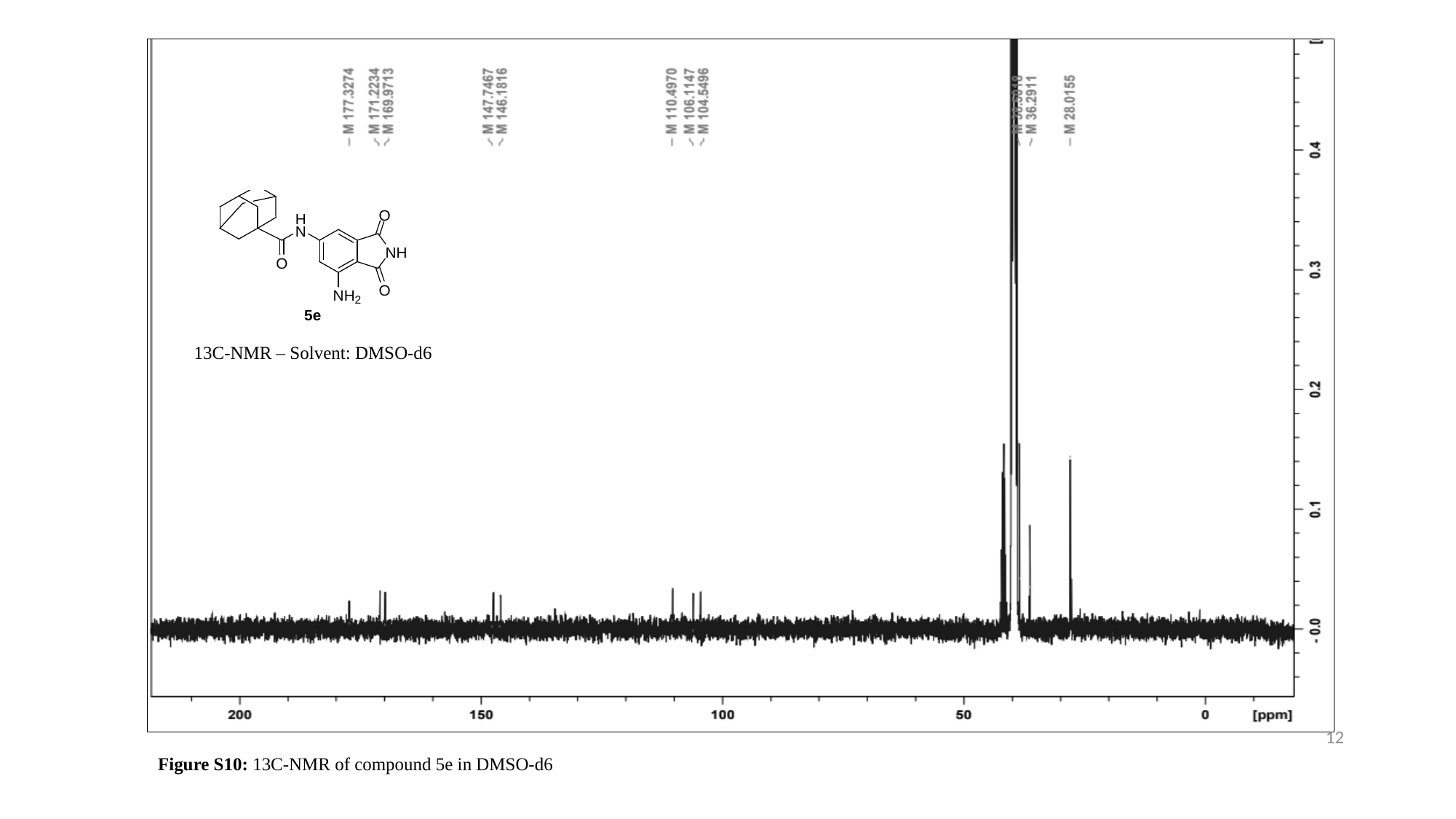

13C-NMR – Solvent: DMSO-d6
12
Figure S10: 13C-NMR of compound 5e in DMSO-d6

## Slide 13
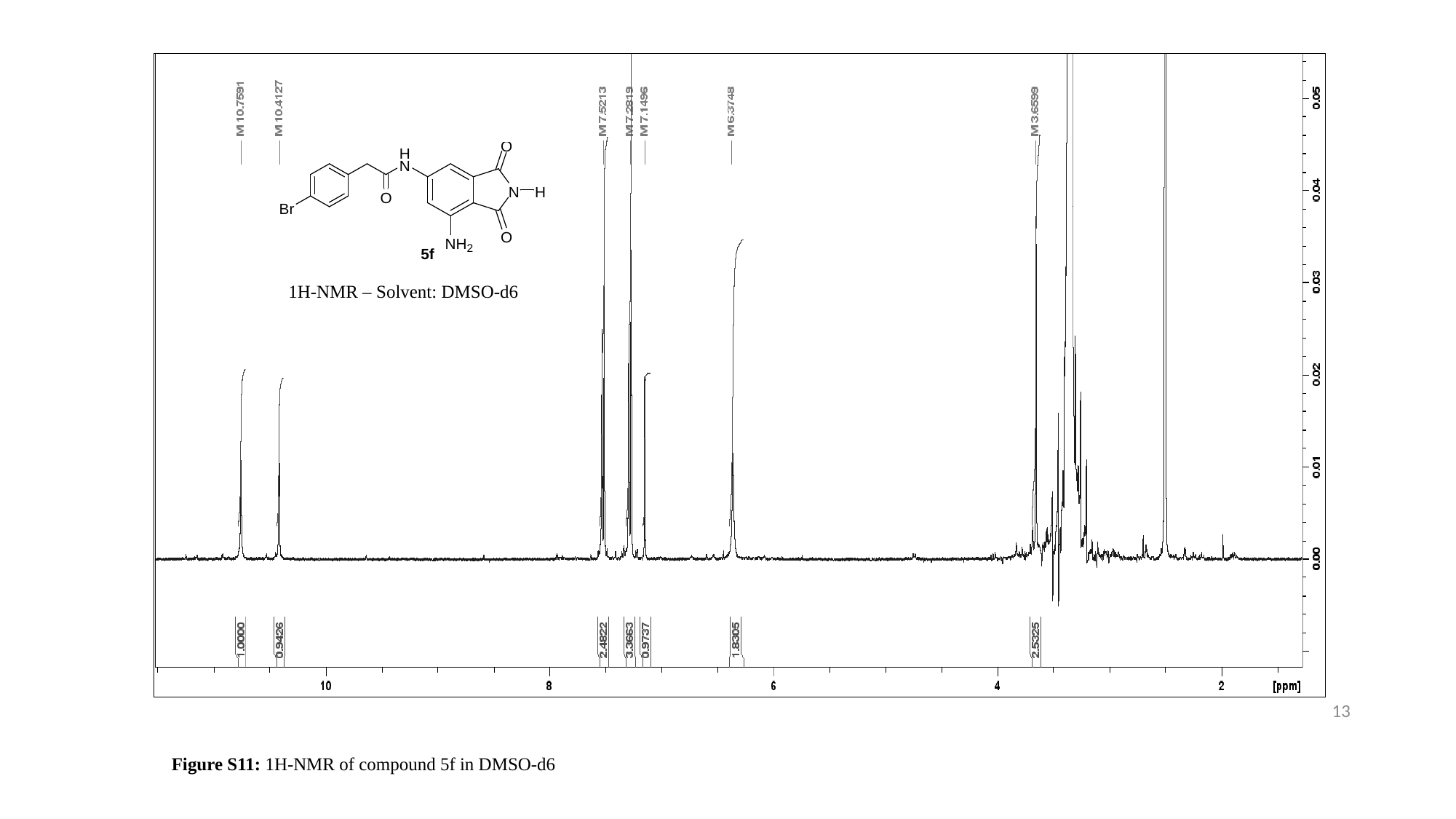

1H-NMR – Solvent: DMSO-d6
13
Figure S11: 1H-NMR of compound 5f in DMSO-d6

## Slide 14
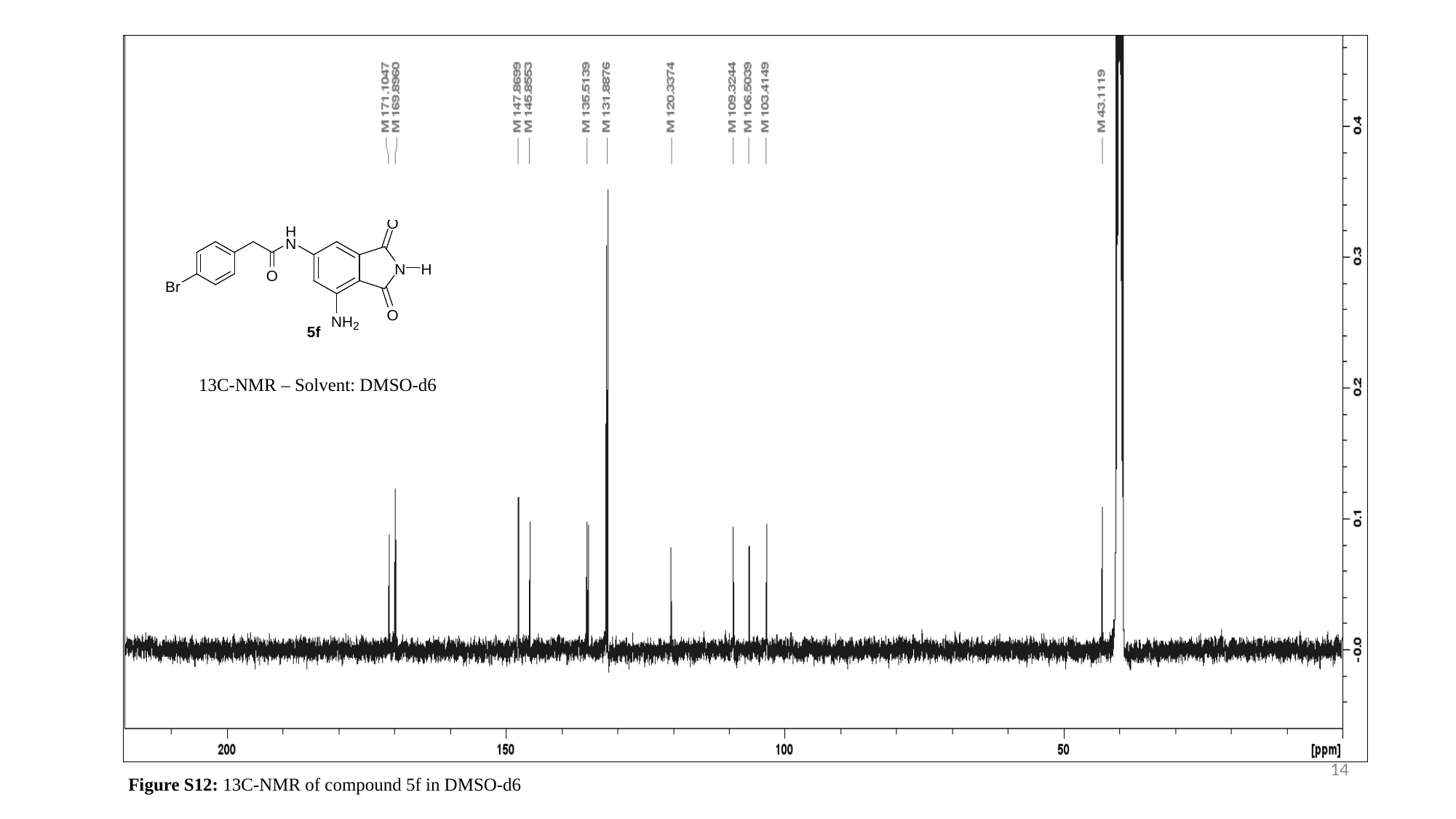

13C-NMR – Solvent: DMSO-d6
14
Figure S12: 13C-NMR of compound 5f in DMSO-d6

## Slide 15
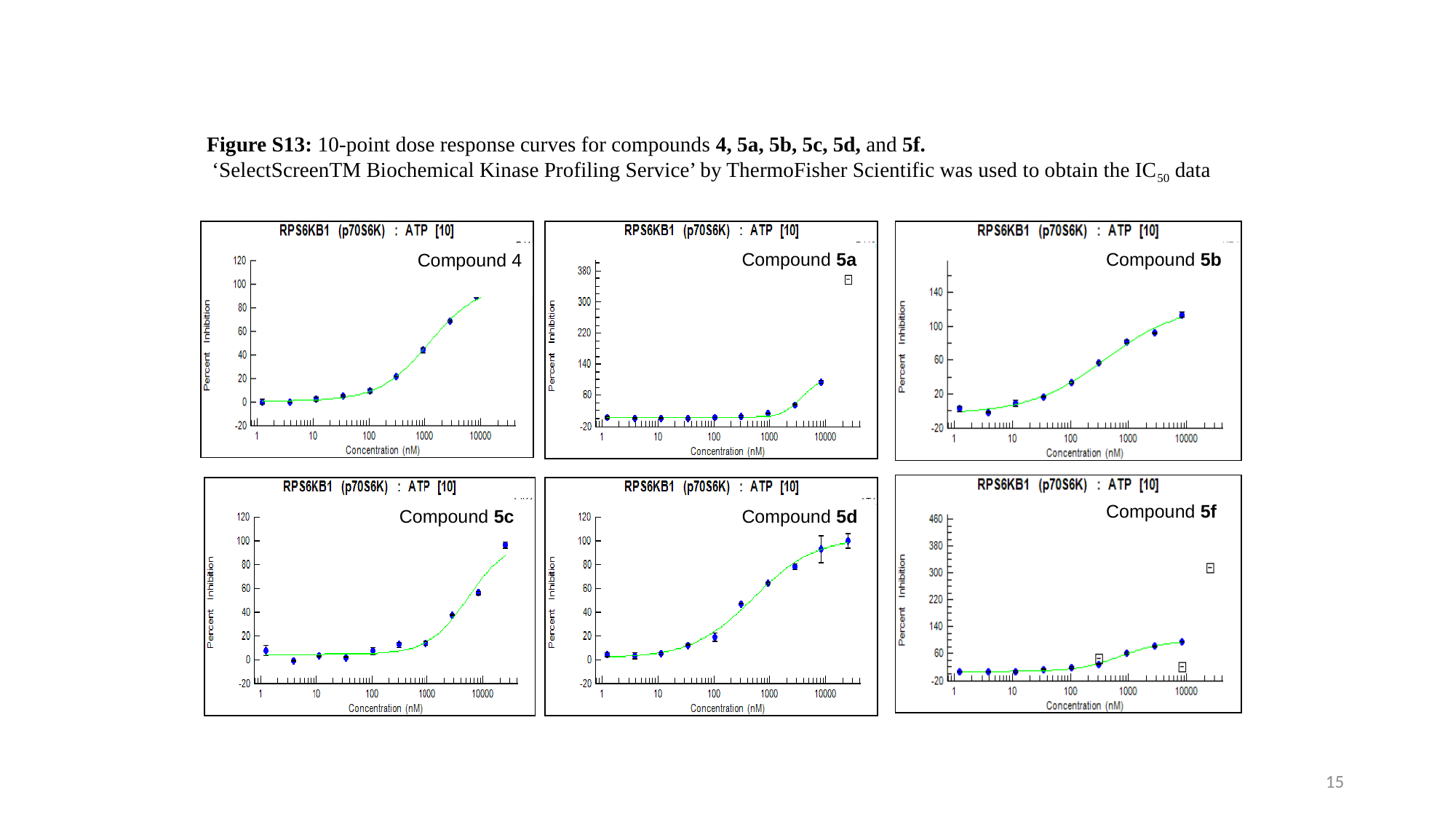

Figure S13: 10-point dose response curves for compounds 4, 5a, 5b, 5c, 5d, and 5f.
 ‘SelectScreenTM Biochemical Kinase Profiling Service’ by ThermoFisher Scientific was used to obtain the IC50 data
Compound 5b
Compound 5a
Compound 4
Compound 5f
Compound 5d
Compound 5c
15

## Slide 16
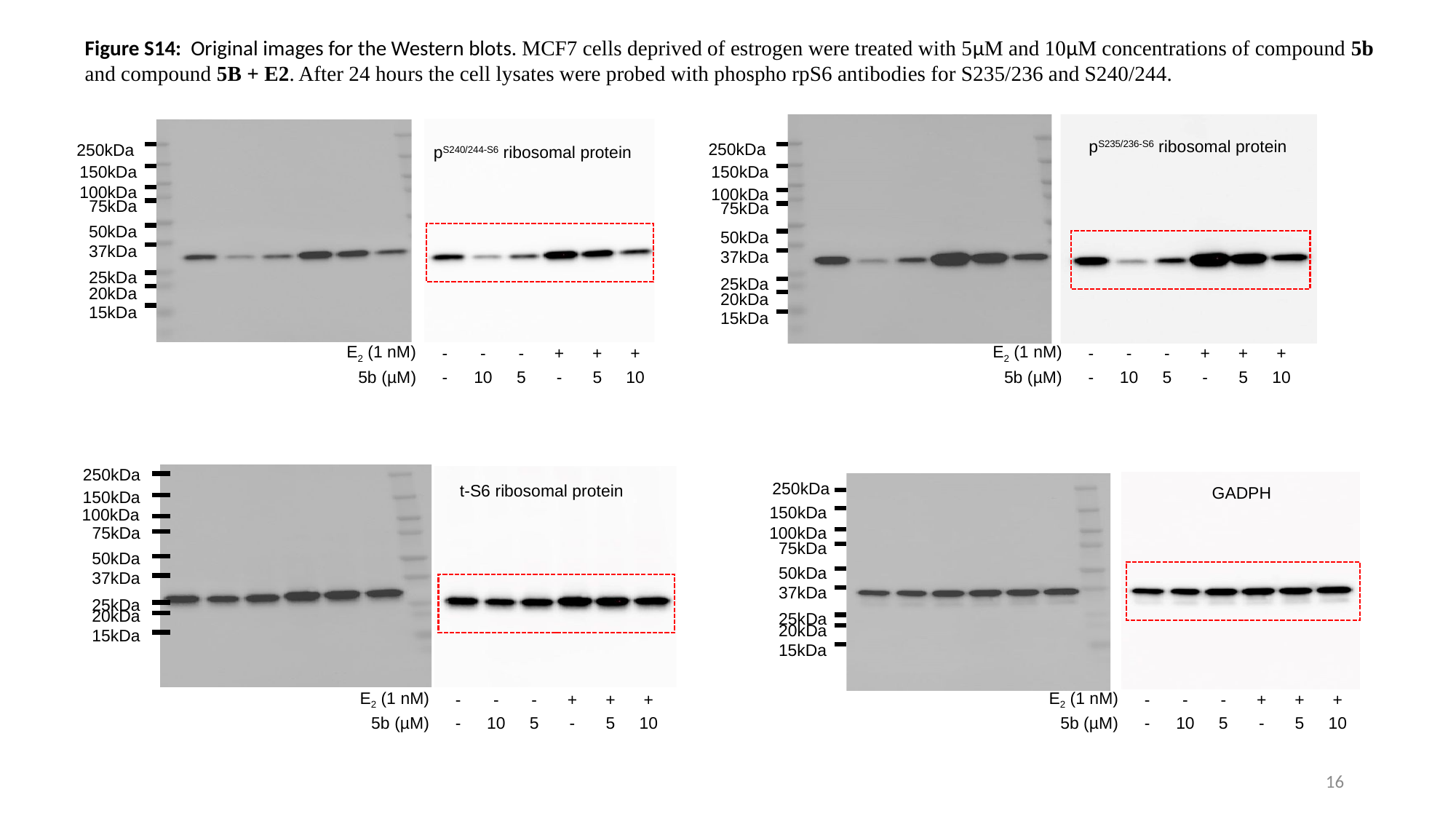

Figure S14:  Original images for the Western blots. MCF7 cells deprived of estrogen were treated with 5μM and 10μM concentrations of compound 5b and compound 5B + E2. After 24 hours the cell lysates were probed with phospho rpS6 antibodies for S235/236 and S240/244.
pS235/236-S6 ribosomal protein
250kDa
250kDa
pS240/244-S6 ribosomal protein
150kDa
150kDa
100kDa
100kDa
75kDa
75kDa
50kDa
50kDa
37kDa
37kDa
25kDa
25kDa
20kDa
20kDa
15kDa
15kDa
| E2 (1 nM) | - | - | - | + | + | + |
| --- | --- | --- | --- | --- | --- | --- |
| 5b (µM) | - | 10 | 5 | - | 5 | 10 |
| E2 (1 nM) | - | - | - | + | + | + |
| --- | --- | --- | --- | --- | --- | --- |
| 5b (µM) | - | 10 | 5 | - | 5 | 10 |
250kDa
250kDa
t-S6 ribosomal protein
GADPH
150kDa
150kDa
100kDa
100kDa
75kDa
75kDa
50kDa
50kDa
37kDa
37kDa
25kDa
20kDa
25kDa
20kDa
15kDa
15kDa
| E2 (1 nM) | - | - | - | + | + | + |
| --- | --- | --- | --- | --- | --- | --- |
| 5b (µM) | - | 10 | 5 | - | 5 | 10 |
| E2 (1 nM) | - | - | - | + | + | + |
| --- | --- | --- | --- | --- | --- | --- |
| 5b (µM) | - | 10 | 5 | - | 5 | 10 |
16

## Slide 17
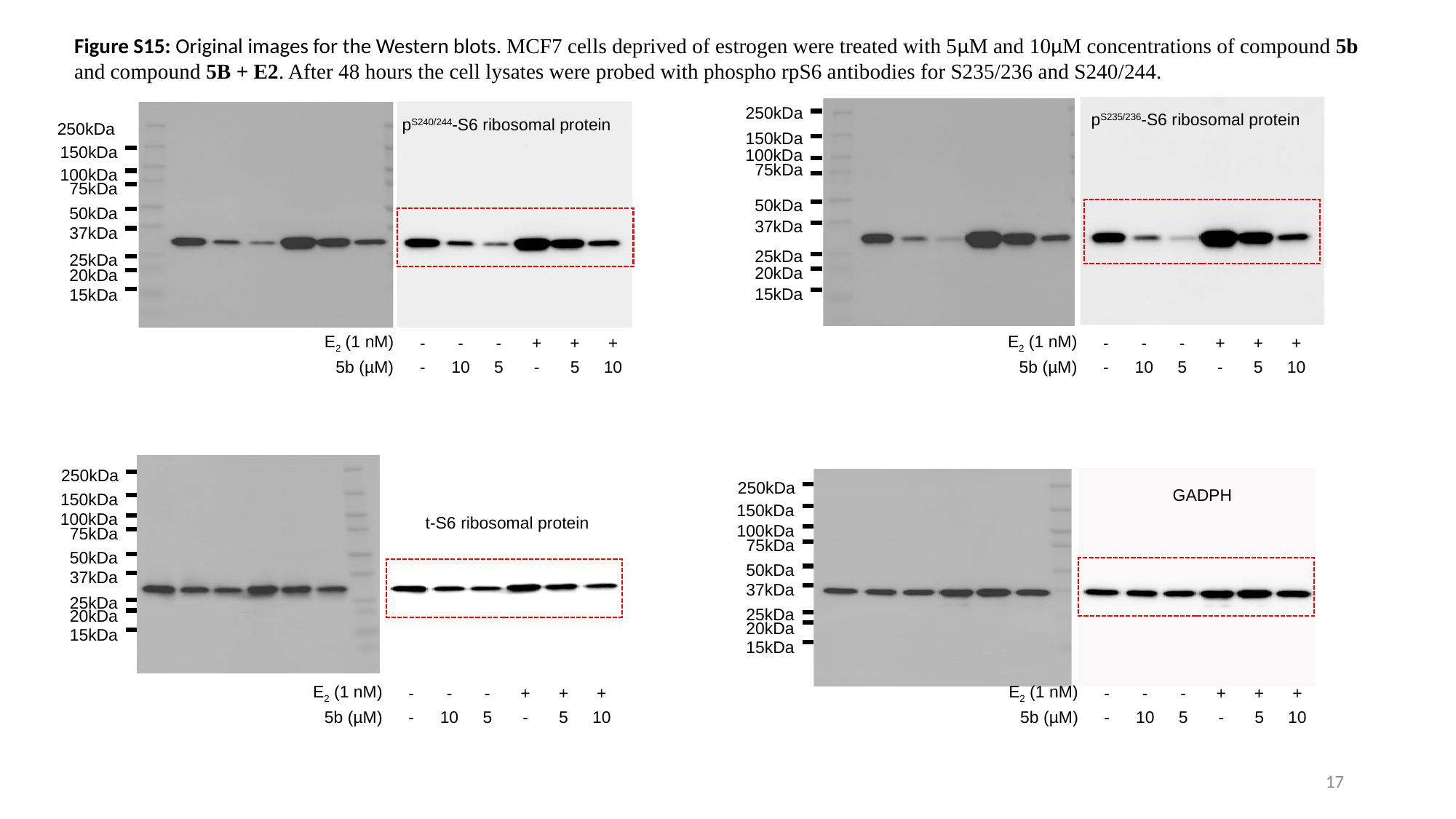

Figure S15: Original images for the Western blots. MCF7 cells deprived of estrogen were treated with 5μM and 10μM concentrations of compound 5b and compound 5B + E2. After 48 hours the cell lysates were probed with phospho rpS6 antibodies for S235/236 and S240/244.
pS235/236-S6 ribosomal protein
150kDa
50kDa
37kDa
25kDa
20kDa
15kDa
250kDa
pS240/244-S6 ribosomal protein
250kDa
150kDa
100kDa
75kDa
100kDa
75kDa
50kDa
37kDa
25kDa
20kDa
15kDa
| E2 (1 nM) | - | - | - | + | + | + |
| --- | --- | --- | --- | --- | --- | --- |
| 5b (µM) | - | 10 | 5 | - | 5 | 10 |
| E2 (1 nM) | - | - | - | + | + | + |
| --- | --- | --- | --- | --- | --- | --- |
| 5b (µM) | - | 10 | 5 | - | 5 | 10 |
250kDa
250kDa
GADPH
150kDa
150kDa
100kDa
t-S6 ribosomal protein
100kDa
75kDa
75kDa
50kDa
50kDa
37kDa
37kDa
25kDa
25kDa
20kDa
20kDa
15kDa
15kDa
| E2 (1 nM) | - | - | - | + | + | + |
| --- | --- | --- | --- | --- | --- | --- |
| 5b (µM) | - | 10 | 5 | - | 5 | 10 |
| E2 (1 nM) | - | - | - | + | + | + |
| --- | --- | --- | --- | --- | --- | --- |
| 5b (µM) | - | 10 | 5 | - | 5 | 10 |
17
